# Gene Loss and Horizontal Gene Transfer Contributed to the Genome Evolution of the Extreme Acidophile “*Ferrovum*”

**DOI:** 10.3389/fmicb.2016.00797

**Published:** 2016-05-31

**Authors:** Sophie R. Ullrich, Carolina González, Anja Poehlein, Judith S. Tischler, Rolf Daniel, Michael Schlömann, David S. Holmes, Martin Mühling

**Affiliations:** ^1^Institute of Biological Sciences, TU Bergakademie FreibergFreiberg, Germany; ^2^Center for Bioinformatics and Genome Biology, Fundación Ciencia & Vida and Depto. de Ciencias Biológicas, Facultad de Ciencias Biológicas, Universidad Andres BelloSantiago, Chile; ^3^Bio-Computing and Applied Genetics Division, Fraunhofer Chile Research Foundation, Center for Systems BiotechnologySantiago, Chile; ^4^Göttingen Genomics Laboratory, Georg-August Universität GöttingenGöttingen, Germany

**Keywords:** acid mine drainage, acidophilic iron oxidizers, “*Ferrovum*”, comparative genomics, genome architecture, mobile genetic elements, horizontal gene transfer, genome evolution

## Abstract

Acid mine drainage (AMD), associated with active and abandoned mining sites, is a habitat for acidophilic microorganisms that gain energy from the oxidation of reduced sulfur compounds and ferrous iron and that thrive at pH below 4. Members of the recently proposed genus “*Ferrovum*” are the first acidophilic iron oxidizers to be described within the *Betaproteobacteria*. Although they have been detected as typical community members in AMD habitats worldwide, knowledge of their phylogenetic and metabolic diversity is scarce. Genomics approaches appear to be most promising in addressing this lacuna since isolation and cultivation of “*Ferrovum*” has proven to be extremely difficult and has so far only been successful for the designated type strain “*Ferrovum myxofaciens*” P3G. In this study, the genomes of two novel strains of “*Ferrovum*” (PN-J185 and Z-31) derived from water samples of a mine water treatment plant were sequenced. These genomes were compared with those of “*Ferrovum*” sp. JA12 that also originated from the mine water treatment plant, and of the type strain (P3G). Phylogenomic scrutiny suggests that the four strains represent three “*Ferrovum*” species that cluster in two groups (1 and 2). Comprehensive analysis of their predicted metabolic pathways revealed that these groups harbor characteristic metabolic profiles, notably with respect to motility, chemotaxis, nitrogen metabolism, biofilm formation and their potential strategies to cope with the acidic environment. For example, while the “*F. myxofaciens*” strains (group 1) appear to be motile and diazotrophic, the non-motile group 2 strains have the predicted potential to use a greater variety of fixed nitrogen sources. Furthermore, analysis of their genome synteny provides first insights into their genome evolution, suggesting that horizontal gene transfer and genome reduction in the group 2 strains by loss of genes encoding complete metabolic pathways or physiological features contributed to the observed diversification.

## Introduction

Acidophilic iron oxidizers represent a phylogenetically diverse group of microorganisms that are physiologically characterized by their ability to use ferrous iron as an electron donor and to thrive at acidic pH (Bonnefoy and Holmes, 2011; Hedrich et al., 2011a; Johnson et al., 2012; Vera et al., 2013; Cárdenas et al., 2016). They often occur in acidic mine effluents and acid mine drainage (AMD) characterized by the acidic pH (< 4) and high metal loads (Schippers et al., 2014).

Members of the novel genus “*Ferrovum*” are the first described acidophilic iron oxidizers within the *Betaproteobacteria* (Hedrich et al., 2011a; Johnson et al., 2014). Based on evidence using molecular biological techniques, members of the genus “*Ferrovum*” have been detected at various mining sites worldwide with different geobiochemical properties regarding pH, dissolved oxygen, and heavy metal ion contents (i.e., Hallberg et al., 2006; Heinzel et al., 2009a; Gonzalez-Toril et al., 2011; Fabisch et al., 2013; Santofimia et al., 2013; Ziegler et al., 2013; Hua et al., 2014; Kay et al., 2014; Jones et al., 2015).

Despite their ubiquitous distribution and their often reported abundance in AMD communities (Hallberg et al., 2006; Heinzel et al., 2009b; Hua et al., 2014; Jones et al., 2015), the isolation and maintenance of “*Ferrovum*” strains in the laboratory have proved to be demanding (Tischler et al., 2013; Johnson et al., 2014). So far only the designated type strain “*Ferrovum myxofaciens*” P3G, which has been retrieved from the mine effluent of the abandoned copper mine Mynydd Parys (Wales, UK), has successfully been isolated, and has been available for a subsequent “classical” physiological characterization (Johnson et al., 2014). The type strain is an autotrophic, psychrotolerant acidophile using ferrous iron as the sole electron donor and oxygen as the sole electron acceptor. It has been observed to produce large amounts of extracellular polymeric substances (Hallberg et al., 2006; Johnson et al., 2014).

Based on phylogenetic analysis the type strain and related clone sequences have been assigned to a novel genus within the *Betaproteobacteria* (Johnson et al., 2014). Although 16S rRNA gene sequence-based analyses have furthermore suggested the existence of several subgroups within the proposed genus “*Ferrovum*” (Tischler et al., 2013; Johnson et al., 2014), their phylogenetic and physiological diversity has not been investigated in further detail due to the unavailability of pure cultures. However, the recent genome study of “*Ferrovum*” sp. JA12 derived from a mine water treatment plant in Lusatia (Tischler et al., 2013) has supported the previous notion that the genus “*Ferrovum*” comprises several subgroups (Ullrich et al., 2016). Initial comparisons to the draft genome of the type strain (Moya-Beltrán et al., 2014) have suggested that both strains represent distinct species within the genus “*Ferrovum*” and that both species are characterized by typical genome properties and nitrogen assimilation strategies (Ullrich et al., 2016). Comparative genomics approaches have not only proved to be extremely useful to investigate the physiological diversity of microorganisms of AMD habitats, but also to explore their genome evolution and mechanisms of speciation (Allen et al., 2007; Andersson and Banfield, 2008; Tyson and Banfield, 2008; Bustamante et al., 2012; Acuña et al., 2013; González et al., 2014; Justice et al., 2014).

Here we report on the findings of a comparative genomics study of a total of four “*Ferrovum*” strains with genome sequences available for analysis: “*F. myxofaciens*” P3G (Moya-Beltrán et al., 2014), “*Ferrovum*” strain JA12 (Mosler et al., 2013; Ullrich et al., 2016), and the “*Ferrovum*” strains PN-J185 and Z-31, which were obtained from the mine water treatment plant in Lusatia and were sequenced as part of this study. Our study provides evidence that connects phylogenetic and physiological diversity among these novel iron oxidizers and uncovers potential driving forces of genome evolution that are hypothesized to have contributed to speciation.

## Materials and methods

### Origin and cultivation of “*Ferrovum*” strains PN-J185 and Z-31

Iron oxidizing mixed cultures were obtained from water samples of the mine water treatment pilot plant in Nochten (Lusatia, Germany; Heinzel et al., 2009a,b) as described previously (Tischler et al., 2013). The microbial composition of mixed cultures was analyzed via terminal restriction fragment length polymorphism and sequencing of 16S rRNA gene fragments as described earlier (Tischler et al., 2013). Fragments of the 16S rRNA gene were amplified and sequenced using the “*Ferrovum*”-specific PCR primer pair 643f (Heinzel et al., 2009b) and fmy_1492r (5′-CTTCACCCCAGTCATGAA-3′). The cultures PN-J185 (Tischler et al., 2013) and Z-31 were found to contain an iron oxidizing strain closely related to “*F. myxofaciens*” P3G and heterotrophic contaminations belonging to the genus *Acidiphilium*. The cultures were periodically transferred into fresh medium and consumption of ferrous iron was used as indicator for the viability of the iron oxidizing “*Ferrovum*” strain. Ferrous iron concentrations were quantified using the ferrozine method (Viollier et al., 2000).

The mixed cultures PN-J185 and Z-31 containing the “*Ferrovum*” strains PN-J185 and Z-31, respectively, were chosen for genome sequencing. The choice was based on their relationship to the previously sequenced “*Ferrovum*” strains “*F. myxofaciens*” P3G (Moya-Beltrán et al., 2014) and “*Ferrovum*” sp. JA12 (Mosler et al., 2013; Ullrich et al., 2016) as suggested by 16S rRNA gene sequence identity. Cells were harvested by centrifugation (5,000 x g) and washed with 50 mM oxalic acid in 0.9% sodium chloride solution. Genomic DNA was extracted from harvested cells using the MasterPure™ Gram Positive DNA Purification Kit (Epicentre Technologies Corp., WI, USA).

During the course of follow-on studies it was noted that “*Ferrovum*” sp. Z-31 and “*Ferrovum*” sp. PN-J185 had apparently been lost within the mixed cultures Z-31 and PN-J185 as was revealed by specific PCR amplification of a 16S rRNA gene fragment. A similar observation has been reported for “*Ferrovum*” sp. JA12 (Ullrich et al., 2016).

### Genome sequencing, assembly, and annotation

The genome of the mixed culture PN-J185 was sequenced via a hybrid approach using the 454 GS-FLX Titanium XL system (titanium GS70 chemistry, Roche Life Science, Mannheim, Germany) and the Genome Analyzer IIx (Illumina, San Diego, CA), while Z-31 was sequenced using Genome Analyzer IIx (Illumina). Paired-end libraries were prepared according to the manufacturer's protocols. Paired-end Illumina reads were pre-processed using Trimmomatic with quality filter Phred 33 (Bolger et al., 2014) resulting in trimmed sequence reads. Sequence reads were mapped to the genome of the contaminating *Acidiphilium* strain (Ullrich et al., 2015) using Bowtie2 allowing only one mismatch (Langmead and Salzberg, 2012). Reads that mapped to *Acidiphilium* spp. were eliminated from the datasets prior to assembly of the genomes of “*Ferrovum*” sp. PN-J185 and “*Ferrovum*” sp. Z-31. The genomes of the “*Ferrovum*” strains were assembled using MIRA (Chevreux et al., 1999, http://sourceforge.net/projects/mira-assembler/) and Newbler 2.8 (Roche Life Science) based on 457,788 454 shotgun reads (genome coverage: 13.8 x) and 4,883,550 112 bp paired-end Illumina reads (genome coverage: 184 x) for PN-J185 and 2,941,282 112 bp paired-end Illumina reads (genome coverage: 133 x) in case of Z-31. Raw data was quality checked and manually inspected using FastQC (Andrews, 2012, http://www.bioinformatics.babraham.ac.uk/projects/fastqc/) and Qualimap (García-Alcalde et al., 2012).

For gap closure of the PN-J185 genome specific primers were designed based on the Staden package GAP4 (The GAP Group, 2012, http://www.gap-system.org) with check of primer specificity via blastn search against the genome. In case of PN-J185 the assembled genome consisted of six contigs and in case of Z-31 of 212 contigs. Previously unassembled sequence reads were mapped to the contigs after gap closure in order to minimize the possibility of missing open reading frames (ORFs) due to assembly artifacts. The vast majority of the before unassembled sequence reads either mapped to the ribosomal RNA gene cluster or to one of the gaps. There was no evidence that one of the assembled contigs may be associated to plasmids.

Gene prediction and automatic annotation were conducted using in-house scripts. Coding sequences were predicted using Prodigal (Hyatt et al., 2010), tRNA genes by tRNAscan-SE (Lowe and Eddy, 1997) and ARAGORN (Laslett and Canback, 2004), and rRNA genes by RNAmmer (Lagesen et al., 2007). The possibility of false-negative gene calls was minimized by manual inspection of larger intergenic regions for missing ORFs. Protein-coding sequences were annotated based on blast searches against downloaded databases of Swiss-Prot, TrEMBL (Boeckmann et al., 2003), and Interpro (Zdobnov and Apweiler, 2001) with an *E*-value cut-off of 1e^−20^ and subsequent filtering for the best hit. Clustered Regularly Interspaced Palindromic Repeats (CRISPRs) were identified using the CRISPR Recognition Tool (CRT, Bland et al., 2007) during further automatic annotation within the pipeline of the integrated microbial genomes/expert review system (IMG/ER, Markowitz et al., 2009, 2012, 2014). Metabolic pathways were deduced by manual inspection of the predicted gene functions based on comparisons to the KEGG database (Kanehisa and Goto, 2000; Kanehisa et al., 2014) and based on characterizations using the NCBI conserved domain search (Marchler-Bauer et al., 2009, 2011, 2015). In case genes of metabolic pathways were found to be absent following pathway reconstruction, the sequence of homologous genes of other bacteria (i.e., *Sideroxydans lithotrophicus, Thiobacillus denitrificans, Gallionella capsiferriformans, Acidithiobacillus ferrooxidans*) was blasted manually against the “*Ferrovum*” genomes. Genes reported to be absent were neither detected using the automatic annotation nor after manual inspection of the annotations.

### Prediction of mobile genetic elements

Insertion elements and transposases in the genome sequences of “*Ferrovum*” sp. PN-J185 and Z-31 and of the type strain “*F. myxofaciens*” P3G (JPOQ01000000) were predicted and classified using TnpPred (Riadi et al., 2012; www.mobilomics.cl/about.html) and ISsaga (Siguier et al., 2006; http://issaga.biotoul.fr/ISsaga/issaga_index.php). Phage-associated genes were predicted by the prophinder algorithm (Lima-Mendez et al., 2008) and blastp searches against the ACLAME database of proteins in viruses and prophages (Leplae et al., 2010; http://aclame.ulb.ac.be/) with the *E*-value cut-off set to 0.001. Putative alien genes and their donors were predicted for “*Ferrovum*” sp. JA12 using Sigi-HMM (Waack et al., 2006) with default settings and were visualized using Artemis (Rutherford et al., 2000). The donor prediction of alien genes was based on comparison with selected codon usage tables (Waack et al., 2006). Thus, taxonomic assignment of alien gene donors is limited to the level of classes.

### Comparative genomics

#### Phylogenomic analysis

Phylogenetic relationship between the “*Ferrovum*” strains was inferred by comparisons of the average nucleotide identity based on blast (ANIb, Goris et al., 2007) and tetranucleotide signature (Richter and Rosselló-Móra, 2009). ANIb and regression of tetranucleotide composition were calculated using the software JSpecies 1.2.1 (Richter and Rosselló-Móra, 2009). Default values of sequence identity cut-off (≥30%), alignment cut-off (≥70%), and query length (1.020 bp) were applied for ANI calculations.

Phylogenetic trees were built based on concatenated alignments of 31 universal protein families (Ciccarelli et al., 2006) of four “*Ferrovum*” strains and selected *Proteobacteria* (Supplementary Table 1). For all bacteria included in the phylogenetic analysis the complete sets of the 31 markers were recovered from their genome sequences. Family members were aligned using MAFFT (Katoh et al., 2002; Katoh and Standley, 2013); the alignments were masked to remove unreliable regions with GBLOCKS (Castresana, 2000), followed by a concatenation of all protein families. Maximum likelihood trees were prepared for concatenated alignments with PhyML (Guindon et al., 2010) with the Whelan and Goldman model (Whelan and Goldman, 2001) to calculate the distance matrix. The evolutionary history was tested using the Boostrap method (Brown, 1994) with 1000 replicates. The analysis was conducted using a Bioperl (Stajich et al., 2002) in-house script.

#### Assignment of protein-coding genes to the COG classification

Protein-coding genes of all four genomes were assigned to the COG classification (Tatusov et al., 2000) by comparison against the COG database using an *E*-value cut-off of 1e^−5^. The association to a COG category was based on the highest hit coverage value using a Bioperl (Stajich et al., 2002) in-house script.

#### Identification of orthologous proteins

The determination of orthologous proteins between the “*Ferrovum*” strains was based on the classification of all protein-coding genes in protein families excluding predicted mobile genetic elements. The selected proteins were sorted using an all-versus-all blastp script based on the Best Bidirectional Blast Hit (BBBH). Protein families were constructed by the 50/50 rule (Snipen and Ussery, 2010: 50% of identity and 50% of coverage in the alignments) and each protein was then assigned to one protein family. The protein families were classified in core-, dispensable-, and unique-genomes according to their distribution across the genomes. The core-genome includes predicted proteins shared by all strains, the dispensable-genome comprises proteins assigned to a subset of strains, and the unique-genome includes proteins assigned to only one single strain. All these subgroups comprise the pan-genome (the union of the genomes under consideration; Tettelin et al., 2005). The determination of the pan-genome was conducted using a Bioperl (Stajich et al., 2002) in-house script at the Center for Systems Biotechnology, Bio-Computing Division and Applied Genetics Division, Fraunhofer Chile Research Foundation (with courtesy of Darwin Guzmán).

#### Comparison and analysis of genome architectures

Blastn-based whole genome comparisons were conducted and visualized using the Blast Ring Image Generator (BRIG, Alikhan et al., 2011) running blast+ version 2.2.30 (Camacho et al., 2009). Pairwise genome comparisons were computed using the online tool DoubleACT (http://www.hpa-bioinfotools.org.uk/pise/double_act.html) running either blastn or tblastx with the *E*-value cut-off set to 0.001. Pairwise comparisons were visualized using the Artemis Comparison Tool (ACT, Carver et al., 2005). Collinear blocks between the genomes were determined within Mauve (Darling et al., 2010; version 2015226, http://darlinglab.org/mauve/mauve.html) with the Match Seed Weight set to 12.

## Results

The comparative genome study involved the type strain “*F. myxofaciens*” P3G (Moya-Beltrán et al., 2014), “*Ferrovum*” sp. JA12 (Ullrich et al., 2016) and the two novel strains Z-31 and PN-J185 which were sequenced as part of the present study. The composite genome of the “*Ferrovum*”-like population FKB7 (Hua et al., 2014) was not included in the study due to lack of information on the level of genome coverage.

### General genome features and phylogenetic relationship of the four “*Ferrovum*” strains

General genome features of the four “*Ferrovum*” strains are shown in Table 1. The type strain “*F. myxofaciens*” P3G (Moya-Beltrán et al., 2014) and “*Ferrovum*” sp. Z-31 have larger genomes (2.70 Mbp and 2.47 Mbp) with higher G+C contents (54.3%) than “*Ferrovum*” sp. JA12 (1.99 Mbp, 44.5%; Ullrich et al., 2016) and “*Ferrovum*” sp. PN-J185 (1.88 Mbp, 39.9%).

**Table 1:** **Comparison of general genome features of four “*Ferrovum*” strains**.

	**“*F. myxofaciens*” P3G^a^**	**“*Ferrovum*” sp**.		
		**Z-31**	**JA12^b^**	**PN-J185**
GenBank Accession number	JPOQ01000000	LRRD00000000	LJWX00000000	LQZA00000000
Genome size (bp)	2,702,191	2,473,037	1,995,737	1,889,241
Number of contigs	647	212	3	6
G+C content (%)	54.3	54.3	44.5	39.9
Number of coding sequences	2859	2492	1970	1888
Number of protein-coding sequences	2785	2303	1882	1844
Number of RNA genes	53	44	43	44
Number of pseudogenes	20	142	45	?

a Moya-Beltrán et al., 2014;

b *Ullrich et al., 2016*.

**Figure 1:**
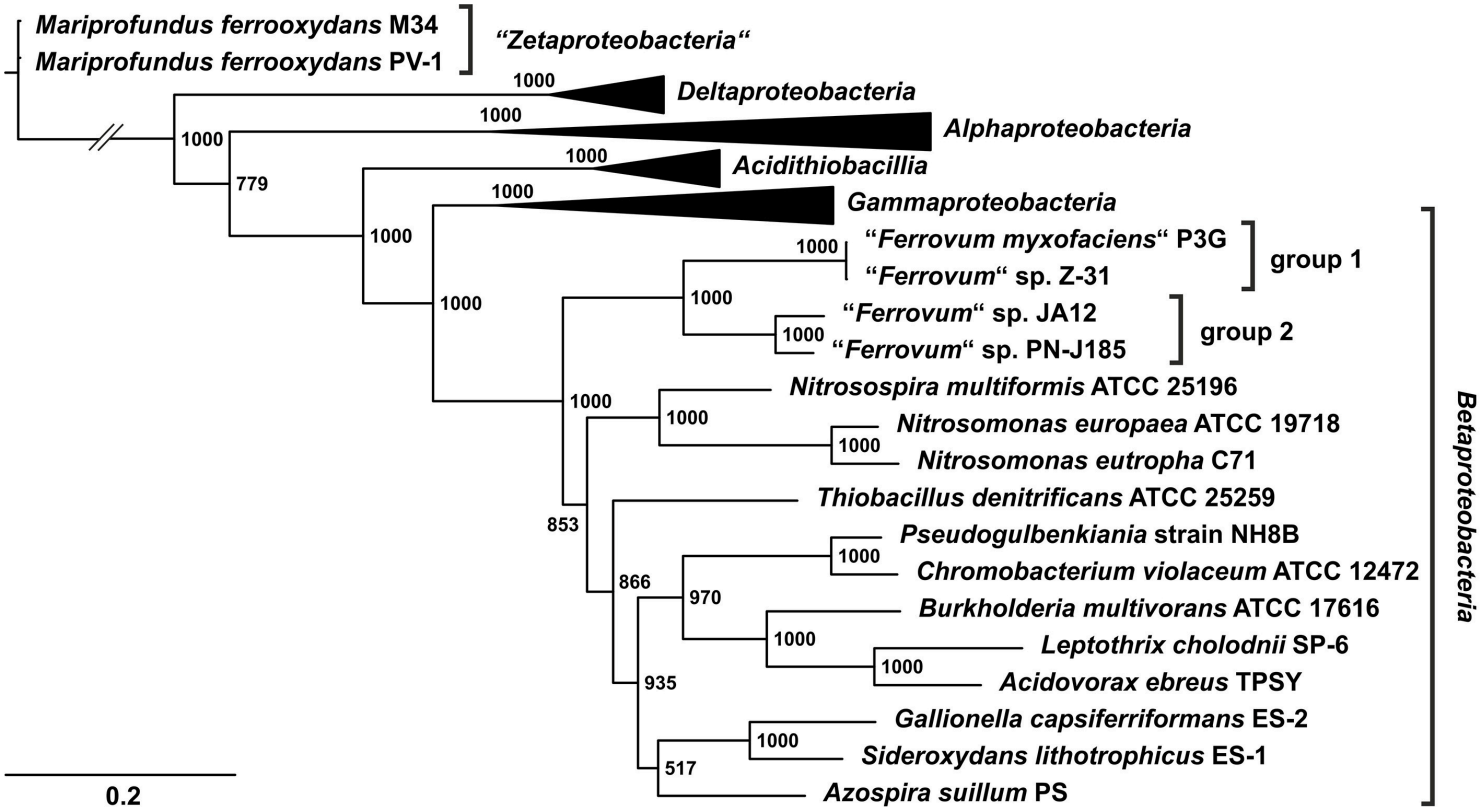
**Phylogenetic tree of the four “*Ferrovum*” strains based on 31 concatenated protein sequences according to (Ciccarelli et al., 2006)**. Concatenated protein sequences of 31 universal markers (Ciccarelli et al., 2006) were aligned using MAFFT (Katoh et al., 2002; Katoh and Standley, 2013). The Maximum likelihood tree was constructed only including informative regions of the multiple sequences alignments using PhyML (Guindon et al., 2010) based on the Whelan & Goldman model (Whelan and Goldman, 2001). Phylogeny was tested using the bootstrap method with 1000 replications. Several subtrees were collapsed. The *Alphaproteobacteria* are represented by *Rhodopseudomonas palustris* TIE-1, *Rhodomicrobium vannielii* ATCC 17100, *Paracoccus denitrificans* PD1222 and *Acidiphilium cryptum* JF-5; the *Deltaproteobacteria* by *Geobacter metallireducens* GS-15 and *Geobacter uraniireducens* Rf4; *Gammaproteobacteria* by *Alcanivorax borkumensis* SK2, *Escherichia coli* K12 substr. MG1655 and *Nitrosococcus oceani* ATCC 19707; the *Acidithiobacillia* by *Acidithiobacillus caldus* SM-1, *Acidithiobacillus ferrivorans* SS3 and *A. ferrooxidans* ATCC 23270. Accession numbers of the genome sequences are given in Supplementary Table 1. The two subbranches harboring the “*Ferrovum*” strains were defined as group 1 and 2.

A dendrogram based on 31 conserved protein sequences selected according to the method of Ciccarelli et al. (2006) was calculated to infer the phylogenetic relationship of the four “*Ferrovum*” strains to other members of the *Proteobacteria* and to each other (Figure 1). This phylogenetic tree supports the previous notion of a 16S rRNA gene sequence-based phylogenetic analysis of “*F. myxofaciens*” P3G and related iron oxidizers with members of the *Nitrosomonadales* being their closest cultivated relatives (Heinzel et al., 2009a; Tischler et al., 2013; Johnson et al., 2014). The four “*Ferrovum*” strains clustered on a distinct branch within the *Betaproteobacteria*, but are separated into two subbranches. The first branch (“*Ferrovum*” group 1) harbors the type strain “*F. myxofaciens*” P3G and “*Ferrovum*” sp. Z-31 while the second branch (“*Ferrovum*” group 2) contains “*Ferrovum*” sp. JA12 and “*Ferrovum*” sp. PN-J185.

Average nucleotide identity (ANI) and the regression of the tetranucleotide composition (tetra) were determined in order to infer the phylogenetic relationships of the four strains (Table 2). The ANIb value of 99.31% and the tetra value of 0.998 strongly indicate that strain Z-31 is very closely related to “*F. myxofaciens*” P3G and also belongs to the type species “*F. myxofaciens*”. The ANIb and tetra values of “*Ferrovum*” sp. JA12 to both “*F. myxofaciens*” strains support the postulation that strain JA12 belongs to a second “*Ferrovum*” species separate from the type species (Ullrich et al., 2016). Based on the phylogenetic indicators “*Ferrovum*” sp. PN-J185 was predicted to represent a third “*Ferrovum*” species which is more closely related to the species represented by “*Ferrovum*” sp. JA12. From here on, the species name “*F. myxofaciens*” will be used instead of “*Ferrovum*” group 1 and “*Ferrovum*” sp. JA12 and “*Ferrovum*” sp. PN-J185 are referred to as group 2 strains JA12 and PN-J185.

**Table 2:** **Genome-based phylogenetic indicators of the four “*Ferrovum*” strains**.

**“*Ferrovum*” sp**.	ANIb (%)^*^				Tetra^*^			
	**P3G^a^**	**Z-31^b^**	**PN-J185^c^**	**JA12^d^**	**P3G**	**Z-31**	**PN-J185**	**JA12**
P3G	100.0	99.31	65.84	66.40	1.000	0.998	0.555	0.611
Z-31		100.0	65.40	65.75		1.000	0.550	0.602
PN-J185			100.0	73.72			1.000	0.951
JA12				100.0				1.000

*The ANIb (blastn-based average nucleotide identity) and tetra (tetranucleotide composition regression) values were calculated using JSpecies (Richter and Rosselló-Móra, 2009)*.

* *Values below the thresholds of ≤ 95% (ANI) and ≤ 0.99 (tetra) indicate that strains belong to different species (Richter and Rosselló-Móra, 2009)*.

a *Percentages of the “F. myxofaciens” P3G genome used for ANIb calculation by pairwise comparison to “Ferrovum” sp. Z-31: 90.8%; to “Ferrovum” sp. PN-J185 22.3%; to “Ferrovum” sp. JA12: 22.5%*.

b *Percentages of the “Ferrovum” sp. Z-31 genome used for ANIb calculation by pairwise comparison to “F. myxofaciens” P3G: 87.3%; to “Ferrovum” sp. PN-J185 23.0%; to “Ferrovum” sp. JA12: 24.5%*.

c *Percentages of the “Ferrovum” sp. PN-J185 genome used for ANIb calculation by pairwise comparison to “F. myxofaciens” P3G: 30.8%; to “Ferrovum” sp. Z-31: 30.0%; to “Ferrovum” sp. PN-J185: 81.9%*.

d *Percentages of the “Ferrovum” sp. JA12 genome used for ANIb calculation by pairwise comparison to “F. myxofaciens” P3G: 29.8%; to “Ferrovum” sp. Z-31: 29.4%; to “Ferrovum” sp. PN-J185: 76.7%*.

### Comparison of inferred metabolic traits

#### Identification of core genes and flexible genes

**Figure 2:**
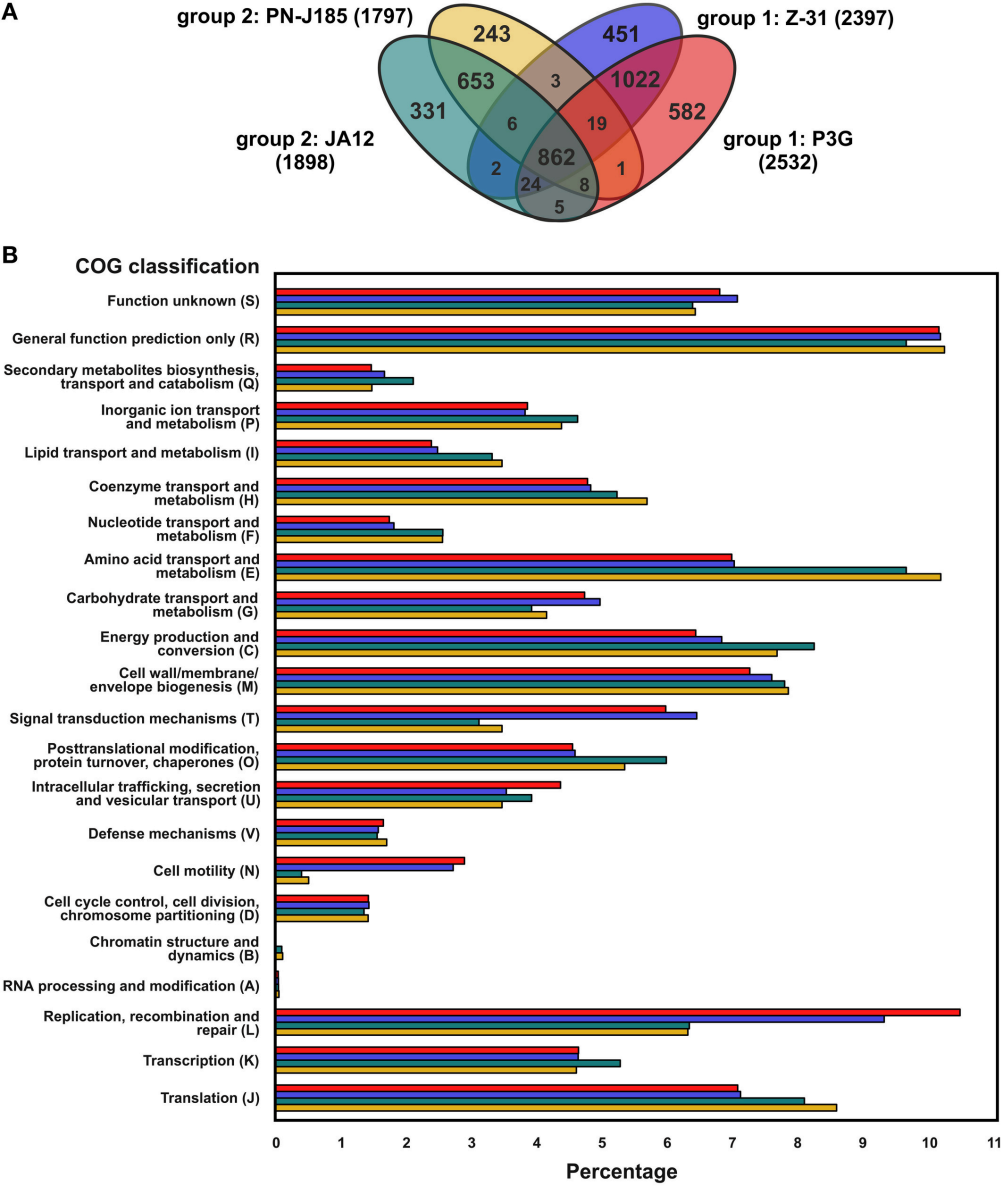
**Summary of homologous proteins of the four “*Ferrovum*” strains and distribution of protein-coding genes assigned to the COG classification. (A)** Core genome and overlaps between the genomes were determined based on the identification of homologous proteins (group 2 strain JA12, turquoise; group 2 strain PN-J185, yellow; group 1 “*F. myxofaciens*” sp. Z-31, blue; group 1 “*F. myxofaciens*” P3G, red). The number of proteins involved in the analysis for each of the genomes is indicated in parentheses. Predicted mobile genetic elements were excluded. **(B)** Percentage of assigned protein-coding genes to COG classes is given for all genomes with color coding according to **(A)**. All protein-coding genes were included in the prediction of the COGs. Results of the predictions are listed in Supplementary Table 2.

The four “*Ferrovum*” strains have a pan-genome of 4213 protein-coding genes and a core genome of 862 protein-coding genes. The non-core genes are flexible genes that are either unique genes only predicted in individual genomes (unique-genome) or genes shared by a subset of the genomes indicated by the overlapping regions of the Venn diagram (dispensable-genome) (Figure 2A). Apart from the core-genome, the genomes of “*F. myxofaciens*” (group 1) and group 2 strains share only few genes suggesting the existence of group-specific metabolic traits.

The assignment of protein-coding genes to the COG classification revealed further insights into the metabolic profiles of the “*Ferrovum*” strains, highlighting the most distinguishing features between the “*F. myxofaciens*” strains and the group 2 strains (Figure 2B and Supplementary Table 2). Differences in the number of assigned protein-coding genes were observed in particular in the COG classes amino acid metabolism and transport (class E), cell mobility (N), signal transduction mechanisms (T), and replication, recombination, repair (L), while the numbers of protein-coding genes assigned to the COG classes with unknown (S) or general predicted functions (R), cell division (D), cell wall/membrane/envelope biogenesis (M), and defense mechanisms (V) were similar in all four genomes.

The metabolic potential of all four strains was subsequently compared at the level of metabolic pathways. In this context, the different number of protein-coding genes assigned to the COG classes E (amino acid metabolism and transport), N (cell mobility), and T (signal transduction mechanisms) was also taken into account.

#### Comparison of the central metabolism

**Figure 3:**
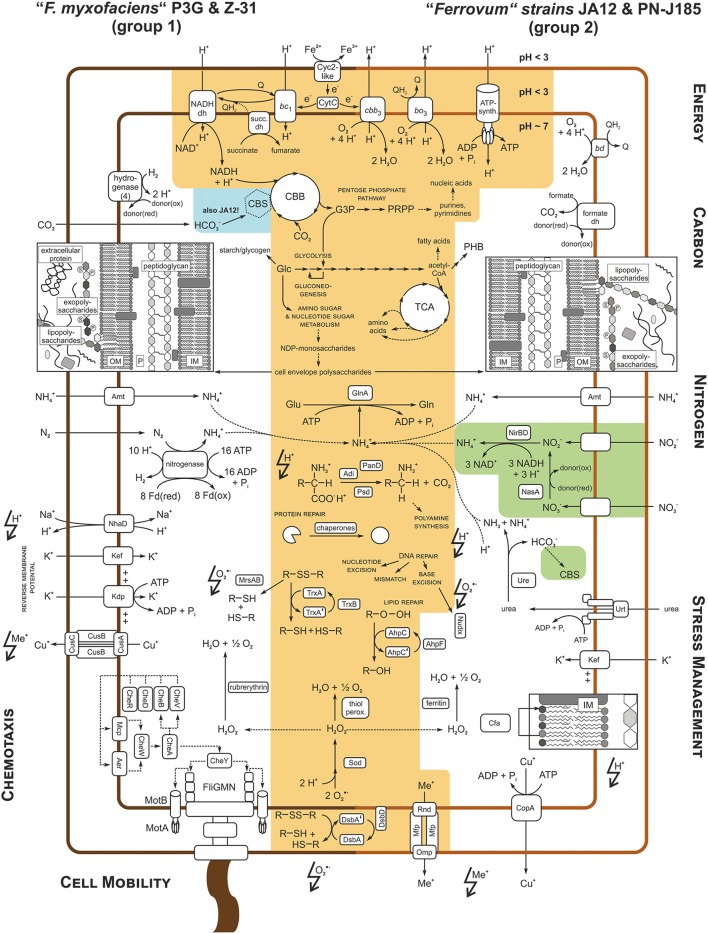
**Comparison of the predicted metabolic potentials of “*F. myxofaciens*” strains P3G and Z-31 and the group 2 strains JA12 and PN-J185**. The comparison of potential metabolic traits was focused on energy, carbon and nitrogen metabolism, strategies of stress management with regard to the chemical constraints of their natural habitats (acidic pH, lightning with proton; oxidative stress, lightning with superoxide radical; high metal loads, lightning with metal ion), as well as chemotaxis and motility. Orange areas show metabolic traits predicted to be shared by all four strains. Metabolic traits only predicted in group 2 strain JA12 are shown in green while traits that were only predicted in the “*F. myxofaciens*” strains and group 2 strain JA12 are colored in blue. For description see text and Supplementary Data 1. Genes predicted to be involved in these metabolic pathways are listed in Supplementary Table 3.

The metabolic potentials of “*F. myxofaciens*” Z-31 and group 2 strain PN-J185 were reconstructed and compared to those of the other strains in order to identify shared metabolic traits and group- and strain-specific features (Figure 3, see also Supplementary Data 1 and Supplementary Table 3). The following section focusses on the main differences in the predicted metabolic profiles between the four “*Ferrovum*” strains.

##### Central carbon metabolism

Similar to “*F. myxofaciens*” P3G (Moya-Beltrán et al., 2014) and the group 2 strain JA12 (Mosler et al., 2013; Ullrich et al., 2016) “*F. myxofaciens*” Z-31 and the group 2 strain PN-J185 were predicted to fix carbon dioxide via the Calvin Benson Bassham cycle (CBB; Figure 3, Supplementary Table 3A). With the exception of group 2 strain PN-J185, the “*Ferrovum*” strains harbor a gene cluster predicted to encode carboxysome shell proteins, a carboxysome-associated carbonic anhydrase and a second ribulose-1,5-bisphosphate carboxylase/oxygenase (RuBisCO) allowing a more efficient carbon dioxide fixation within a carboxysome (CBS; Cannon et al., 2001). The carbon fixation product 3-phosphoglycerate (G3P) was predicted to be converted to the precursors for the biosynthesis of amino acids, fatty acids, polysaccharides, carbon storage compounds, and nucleic acids via the pathways of the central metabolism.

The conversion of G3P by gluconeogenesis was predicted to lead to the formation of glucose-6-phosphate and glucose-1-phosphate, the major precursors for biosynthesis of cell envelope polysaccharides. But apparently, only the “*F. myxofaciens*” strains are also able to phosphorylate glucose directly via glucokinase activity (not shown in Figure 3). However, all “*Ferrovum*” strains share a core set of genes predicted to be involved in amino and nucleotide sugar metabolism in which glucose-1-phosphate and glucose-6-phosphate are converted to precursors for the biosynthesis of the cell envelope polysaccharides peptidoglycan, lipopolysaccharides and, potentially, also of exopolysaccharides (Table 3). The “*F. myxofaciens*” strains harbor a slightly larger enzyme repertoire potentially enabling them to produce also ADP-glucose and CDP-4-keto-3, 6-desoxy-D-glucose as alternative precursors. Furthermore, “*F. myxofaciens*” P3G and the group 2 strain PN-J185 are predicted to share the potential to convert GDP-mannose to GDP-4-keto-D-rhamnose, but only group 2 strain PN-J185 appears to catalyze the further conversion to D-fucose.

**Table 3:** **Potential precursors for the biosynthesis of polysaccharides in the four “*Ferrovum*” strains**.

**Nucleotide-activated monosaccharide**	**“*****F. myxofaciens*****”**		**Group 2 strain**	
	**P3G**	**Z-31**	**JA12**	**PN-J185**
UDP-glucose	+	+	+	+
UDP-glucuronate	+	+	+	+
UDP-N-acetylglucosamine	+	+	+	+
ADP-glucose	+	+	-	-
CDP-glucose	-	+	-	-
CDP-4-dehydro-6-desoxy-glucose	-	+	-	-
CDP-4-dehydro-3,6-didesoxy-glucose	(+)	+	(+)	(+)
TDP-glucose	+	+	+	+
TDP-4-dehydro-6-desoxy-glucose	+	+	+	+
UDP-galactose	+	+	+	-
UDP-galacturonate	+	+	+	+
UDP-N-acetylgalactosamine	+	+	-	-
UDP-N-acetylgalactosaminuronic acid	+	+	(+)	(+)
UDP-mannose	+	+	+	+
UDP-mannosamine	+	+	+	-
GDP-4-dehydro-6-desoxy-mannose	+	-	-	+
TDP-rhamnose	+	+	+	+
TDP-4-dehydrorhamnose	+	+	+	+
GDP-fucose	-	-	-	+
UDP-N-acetyl-muramate	+	+	+	+

*The predicted enzyme repertoire of the amino and nucleotide sugar metabolism may lead to the formation of a variety of nucleotide-activated monosaccharides as potential precursors for the biosynthesis of cell envelope polysaccharides. The genes predicted to be involved are listed in Supplementary Table 3A*.

*+ Required genes for the formation are present; (+) set of genes required for the formation appears to be incomplete; -required genes for the formation were not predicted*.

All four strains were also found to harbor a core set of homologous glycosyltransferases potentially involved in the synthesis of the cell envelope polysaccharides. However, the presence of additional glycosyltransferases that were predicted to be unique for “*F. myxofaciens*” strains or for either of the group 2 strains suggests the production of lipo- and exopolysaccharides with group- and strain-specific monosaccharide compositions (Supplementary Table 3A). The cell envelope polysaccharides may be exported via the ABC-2-type polysaccharide transporter or via the Lpt-type lipopolysaccharide export system that were identified in all strains. Taken together the shared gene repertoire potentially enables all “*Ferrovum*” strains to produce exopolysaccharides as components of biofilms. But only the “*F. myxofaciens*” strains harbor genes predicted to encode the PEP-CTERM exosortase system which is presumed to be involved in protein export during biofilm formation in other bacteria (Haft et al., 2006; Craig et al., 2011). Similar to other bacteria the genes encoding the PEP-CTERM exosortase system are co-localized with genes predicted to be involved in the synthesis and export of exopolysaccharides (Supplementary Table 3A).

In contrast to other (acidophilic) chemolithoautotrophs (Cárdenas et al., 2010) all “*Ferrovum*” strains were predicted to exhibit the complete set of enzymes of the tricarboxylic acid cycle (TCA) (Mosler et al., 2013; Moya-Beltrán et al., 2014; Ullrich et al., 2016). Although a complete TCA is thought to indicate a heterotrophic lifestyle (Wood et al., 2004), no uptake systems for organic carbon compounds were identified in the “*Ferrovum*” genomes apart from several amino acid ABC transport systems and the urea ABC transporter in the group 2 strains. Notably, the genomes of the group 2 strains were predicted to encode a higher copy number of these amino acid ABC transporters explaining the higher percentage of protein-coding genes assigned to the COG class E (Figure 2B).

The fixed carbon compounds may also be converted to storage compounds. While the “*F. myxofaciens*” strains appear to store fixed carbon compounds in glucose-based polymers such as starch or glycogen, the group 2 strains harbor genes associated with the synthesis and hydrolysis of polyhydroxybutyrates (Figure 3).

##### Nitrogen metabolism

The comparison of the metabolic profiles of the four “*Ferrovum*” strains revealed variations with respect to their nitrogen metabolism (Figure 3, Supplementary Table 3B). Although all four strains share the potential to take up ammonium via an Amt family transporter and to transfer it to glutamate using a glutamine synthetase, they differ with regard to the utilization of alternative nitrogen sources to ammonium.

Similar to “*F. myxofaciens*” P3G (Moya-Beltrán et al., 2014) the genome of “*F. myxofaciens*” Z-31 harbors the set of genes required for the fixation of molecular nitrogen via nitrogenase. Group 2 strain PN-J185 in contrast, shares the potential to utilize urea via a urea ABC transporter and the urea-hydrolyzing enzyme urease with the group 2 strain JA12 (Mosler et al., 2013; Ullrich et al., 2016). While both strains may utilize ammonium derived from the hydrolysis of urea as nitrogen source, only group 2 strain JA12 may also utilize the released bicarbonate as carbon source via carbon fixation in the carboxysome.

The potential to reduce nitrate to ammonium is unique for the group 2 strain JA12 (Mosler et al., 2013; Ullrich et al., 2016) since genes encoding transporters and assimilatory reductases for nitrate or nitrite were not identified in the other genomes. However, homologous genes were identified in the “*Ferrovum*”-like population FKB7 (Hua et al., 2014).

##### Energy metabolism

All “*Ferrovum*” strains harbor homologous redox proteins predicted to be involved in ferrous iron oxidation and electron transfer to the terminal electron acceptors NAD(P)^+^ (uphill) and oxygen (downhill). Thus, all strains potentially employ similar electron transfer processes from ferrous iron to the terminal acceptors to those proposed for the group 2 strain JA12 (Ullrich et al., 2016) and for the well-studied model acidophile *A. ferrooxidans* (Valdés et al., 2008). That is, a Cyc2-like high molecular mass cytochrome predicted to oxidize ferrous iron in the outer membrane and soluble *c*-type cytochromes assumed to transfer the electrons through the periplasm to the respiratory complexes in the inner membrane (Figure 3, Supplementary Table 3C).

While genes encoding the terminal oxidases *cbb*_3_-type cytochrome *c* oxidase and *bo*_3_-type quinol oxidase were predicted in all four strains, only the group 2 strains also encode the *bd*-type quinol oxidase which is characterized by its high affinity to oxygen (Borisov et al., 2011).

Similar to group 2 strain JA12 (Ullrich et al., 2016) the genome of group 2 strain PN-J185 encodes a formate dehydrogenase of unknown physiological relevance. In contrast to that, the genomes of the “*F. myxofaciens*” strains were found to contain a gene cluster similar to the one in *A. ferrooxidans* ATCC 23270 predicted to encode a hydrogen-evolving hydrogenase (Valdés et al., 2008).

##### Cell mobility and chemotaxis

Apparently, the differences in the number of genes assigned to the COG classes N (cell mobility) and T (signal transduction) is based on the lack of genes predicted to be involved in flagella formation and chemotaxis in the group 2 genomes (Figure 3, Supplementary Table 3D). The genomes of the “*F. myxofaciens*” strains contain 47 genes associated with the formation of the flagellum apparatus and 16 genes encoding two-component signal transduction systems (chemotaxis). The presence of these genes suggests that the “*F. myxofaciens*” strains have the ability to actively move in response to environmental stimuli.

#### Diversity of predicted stress tolerance mechanisms

The comparison of the metabolic profiles of the four “*Ferrovum*” strains was extended to potential strategies to cope with the chemical constraints of their natural habitats including acidic pH, high metal ion concentrations and oxidative stress. Again all strains share a core set of genes potentially involved in stress management strategies, while other features appear to be group-specific (Figure 3, Supplementary Table 3E).

##### Maintaining the intracellular pH homeostasis

Uncontrolled influx of protons due to the natural proton concentration gradient across the membrane may cause severe disturbances of the intracellular pH homeostasis. Like many acidophiles all “*Ferrovum*” strains seem to prevent the uncontrolled proton influx by generating a reversed (inside positive) membrane potential that is believed to be achieved via the increased uptake of potassium ions (Baker-Austin and Dopson, 2007; Slonczewski et al., 2009; Chen et al., 2015). While all “*Ferrovum*” strains exhibit the predicted Kef-type potassium transporters, only the “*F. myxofaciens*” strains additionally harbor the Kdp-type potassium uptake ATPase (Figure 3, Supplementary Table 3E).

To cope with an increase of the intracellular proton concentration all “*Ferrovum*” strains can export excess protons via the respiratory complexes driven by downhill electron transfer. The “*F. myxofaciens*” strains appear to also use Na^+^/H^+^-antiporters (NhaD) which were not detected in the group 2 strains.

Genes for buffering of the intracellular pH by decarboxylation of amino acids and synthesis of polyamines were detected in all four “*Ferrovum*” strains, but only the group 2 strains harbored genes for buffering capacity using ammonia derived from urea hydrolysis. The role of the urease in the pH homeostasis has originally been shown for the gastric pathogen *Helicobacter pylori* (Eaton et al., 1991; Sachs et al., 2005), but has also been suggested for group 2 strain JA12 (Ullrich et al., 2016) and for *Thiomonas* sp. CB2 (Farasin et al., 2015).

##### Coping with high metal loads

The identification of several gene clusters encoding predicted RND (resistance-nodulation-cell division protein) pumps, membrane fusion proteins (MFP), and outer membrane proteins (Omp) in each of the genomes suggests that all “*Ferrovum*” strains have the capacity to use cation/multidrug transporters of the RND family to cope with high metal ion concentrations as described in other bacteria (Kim et al., 2011) (Figure 3, Supplementary Table 3E). However, both “*Ferrovum*” groups were predicted to employ different systems promoting the efflux of copper ions. While the “*F. myxofaciens*” strains harbor a complete Cus system (Kim et al., 2011) consisting of a pump (CusA), the channel-forming membrane fusion proteins (CusB), the outer membrane protein (CusC) and the small periplasmic protein CusF, the group 2 strains seem to use the copper-exporting ATPase CopA instead.

##### Oxidative stress management

Oxidative stress is mediated via reactive oxygen species (Imlay, 2013) generated by iron-catalyzed Haber-Weiss reactions (Haber and Weiss, 1934) and the Fenton reaction (Walling, 1975). Although all strains were predicted to detoxify superoxide radicals using superoxide dismutase, they appear to scavenge the resulting hydrogen peroxide in different ways (Figure 3, Supplementary Table 3E). The “*F. myxofaciens*” strains are predicted to use rubrerythrin to convert hydrogen peroxide to water and oxygen as reported in many other acidophiles (Cárdenas et al., 2012), while the group 2 strains appear to use thiol peroxidases or ferritins.

All “*Ferrovum*” strains have the capacity to use the thioredoxin (TrxA)/thioredoxin reductase (TrxB)-dependent system to repair oxidatively damaged cytoplasmic proteins and the thiol:disulfide interchange proteins DsbA and DsbD to repair periplasmic proteins. However, only the “*F. myxofaciens*” strains are predicted to be able to repair oxidatively damaged methionine residues since a methionine sulfoxide reductase (MrsAB) was identified only in the genomes of “*F. myxofaciens*” P3G and Z-31.

The redox state of oxidatively damaged lipids may be restored via the peroxiredoxin (AhpC)/alkyl hydroxide peroxidase (AhpF)-dependent system in all strains. In the genomes of group 2 six copies of *ahpC* were identified, while the genomes of “*F. myxofaciens*” contain three copies of the peroxiredoxin encoding gene. The group 2 strains may additionally use a glutathione peroxidase to cope with damaged lipids based on the identification of genes predicted to be involved in the biosynthesis of glutathione and the glutathione peroxidase. However, it remains unclear how the group 2 strains recycle oxidized glutathione since a glutathione disulfide reductase was not detected in their genomes.

Although the repertoire of genes putatively involved in oxidative stress management varies slightly between the “*Ferrovum*” groups, all strains appear to exhibit similar strategies to cope with oxidative stress and oxidatively damaged biomass.

### Identification of potential driving forces of genome evolution

Genome evolution can be driven by the acquisition of genes, which is often accompanied by events of horizontal gene transfer (HGT) and the loss of genes and genome segments (Gogarten et al., 2002; Boon et al., 2014). HGT is facilitated by mobile genetic elements and often leaves signatures in the genome including integration sites (often associated with tRNA genes), abnormal G+C contents or varied codon usage (Waack et al., 2006; Juhas et al., 2009; Bustamante et al., 2012; Acuña et al., 2013). The comparison of the inferred metabolic profiles revealed distinguishing metabolic traits between both “*Ferrovum*” groups and also between both group 2 strains. Relating the identified differences of their metabolic profiles to their genome architectures allowed the deduction of mechanisms that may have contributed to genome evolution and diversification of the “*Ferrovum*” strains.

To identify such mechanisms, mobile genetic elements were first predicted and classified in the four genomes (section Prediction of Mobile Genetic Elements). Then regions of interest, such as unique regions in a subset of genomes or regions with abnormal G+C contents, were identified by blastn-based whole genome comparisons using BRIG (Alikhan et al., 2011). Each genome was used in turn as a reference genome for the blastn-based whole genome comparison and blastn matches to the reference genome were plotted together with the G+C content of the reference (section Linking the Differences in the Predicted Metabolic Profiles to the Genome Architectures). Regions of interest were analyzed in more detail using the synteny viewers Mauve (Darling et al., 2010) and the Artemis Comparison Tool (ACT, Carver et al., 2005) and were inspected for the presence of signatures of genomic islands, mobilization (presence of transposases, integrases, phage-associated genes, integration sites) and conjugation (type IV secretion system). While Mauve (Darling et al., 2010) identifies conserved DNA segments in the compared genomes (collinear blocks), ACT allows a more detailed comparison at the sequence level based on blast (section Linking the Differences in the Predicted Metabolic Profiles to the Genome Architectures).

#### Prediction of mobile genetic elements

Transposases and integrases were predicted to be present in all “*Ferrovum*” genomes and were classified using TnpPred (Riadi et al., 2012) and ISsaga (Siguier et al., 2006) (Supplementary Table 4). Many more transposable elements were predicted in the “*F. myxofaciens*” genomes (Z-31: 84, P3G: 207) compared to those of the group 2 strains (JA12: 35, PN-J185: 35). While transposable elements of IS classes IS3, ISL3, IS21 and IS200 are present in all four genomes, there are also IS classes that were only detected in the genomes of individual strains; such as IS10 in group 2 strain JA12, or IS66 and IS110 in “*F. myxofaciens*” Z-31.

**Figure 4:**
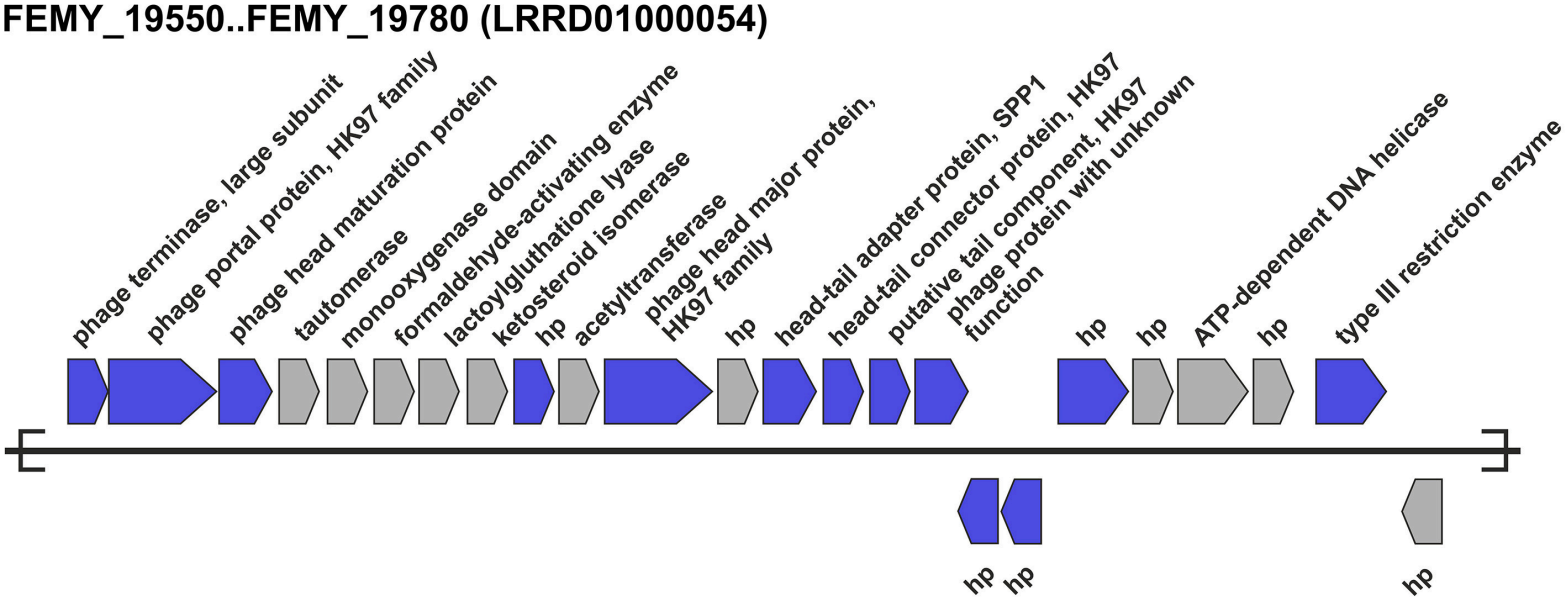
**Predicted prophage fragment in the genome of “*F. myxofaciens*” Z-31**. One contig of the “*F. myxofaciens*” Z-31 genome was found to harbor a cluster of predicted phage-associated genes (blue) which were identified using Prophinder (Lima-Mendez et al., 2008) by conducting blastp searches against the ACLAME database (Leplae et al., 2010). Genes encoding hypothetical proteins (hp) and genes that are not predicted to be associated with phages are shown in gray. The nucleotide accession number of the contig is given in parentheses.

The presence of predicted phage-associated genes in all “*Ferrovum*” genomes indicates that they may have been targets of phage infections (Supplementary Table 4). Predicted phage-associated genes were found to be scattered throughout the genomes with the exception of “*F. myxofaciens*” Z-31, where several phage-associated genes were clustered on a small contig (Figure 4). This gene cluster appears to be a fragment of a prophage. It harbors genes predicted to be associated with the phage DNA packaging machinery (large subunit of the phage terminase, portal protein) and phage head and tail formation (head maturation protein, head-to-tail adapter protein, tail protein). Other characteristic functions associated with prophages (small subunit of the terminase, phage DNA replication and cell lysis; Canchaya et al., 2003) were not identified in the genome of “*F. myxofaciens*” Z-31. Based on comparison to the ACLAME database, the prophage fragment was found to encode hypothetical proteins related to phages in other bacteria. No potential phage integration sites were identified since predicted phage DNA integrases and recombinases were located on other contigs not associated with the prophage fragment.

All “*Ferrovum*” genomes were furthermore found to harbor signatures of conjugation based on the identification of a nearly complete set of the type IV secretion system, which is homologous to the VirB/D4 secretion system in *Agrobacterium tumefaciens* (Wallden et al., 2010 and Supplementary Table 4). However, genes encoding VirB1 and VirB7 are absent in all of the “*Ferrovum*” genomes and the genomes of the “*F. myxofaciens*” strains also lack the gene encoding protein D4.

Genes predicted to encode plasmid stabilization system proteins were identified in the genomes of “*F. myxofaciens*” P3G and “*F. myxofaciens*” Z-31 (Supplementary Table 4). The genomes of “*F. myxofaciens*” P3G and “*Ferrovum*” sp. JA12 were also predicted to encode a plasmid segregation oscillating ATPase and a plasmid segregation centromere-binding protein. Although homologous proteins are known to form the ParAB partition system which is involved in segregation of low copy number plasmids, it is also involved in bacterial chromosome segregation (Lioy et al., 2015). No further evidence was found for the presence of plasmids (i.e., potential plasmid-associated contigs) in any of the “*Ferrovum*” strains.

#### Linking the differences in the predicted metabolic profiles to the genome architectures

**Figure 5:**
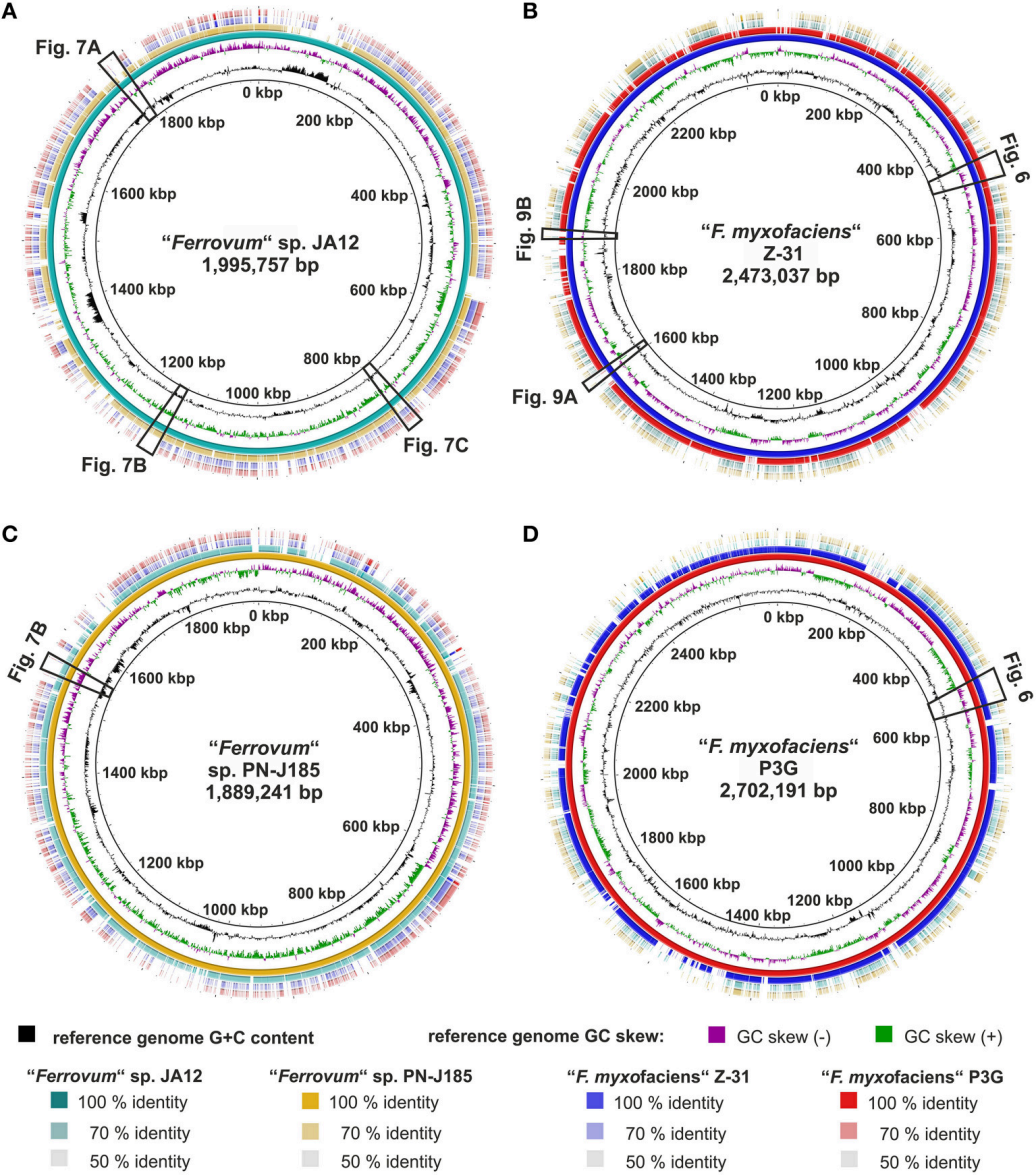
**Reference-based whole genome comparison of the four “*Ferrovum*” strains**. Blastn-based whole genome comparison and visualization were performed using BRIG (Alikhan et al., 2011) with each of the four genomes used as a reference in turn. The reference genome and its size are indicated in the figure center: **(A)** group 2 strain JA12, **(B)** “*F. myxofaciens*” Z-31, **(C)** group 2 strain PN-J185, **(D)** “*F. myxofaciens*” P3G. For the reference genome also G+C content and GC skew are shown on the first and the second ring from the inside. Circular representation of the reference genomes (third ring) is based on the concatenation of the contigs. Contigs of group 2 strain JA12 were ordered FERRO_contig000001 to FERRO_contig000003 (LJWX01000001 to LJWX01000003), while contigs of the other strains were ordered randomly. Matches to the reference genomes are displayed color-coded (JA12, turquoise; PN-J185, orange; Z-31, blue; P3G, red) on the rings four to six. The levels of the match sequence identities are indicated by color intensity (the higher the color intensity the higher the sequence identity). Cross references link to other figures showing a more detailed analysis of regions of interest.

Blastn-based whole genome comparisons were conducted and visualized using BRIG (Figures 5A–D). The notion that strains belonging to the same group share more similar metabolic profiles was further underlined by the presence or absence of the corresponding genes and by the level of sequence identity of these genes in the various strains. The sequence identities of up to 100% between both “*F. myxofaciens*” genomes suggest a very close relationship of both “*F. myxofaciens*” strains (Figures 5B,D). In the group 2 genomes, the identities of matches (Figures 5A,C) are considerably lower supporting the hypothesis that both group 2 strains now represent distinct “*Ferrovum*” species as indicated by the ANIb and tetra values (Table 2).

##### Gene cluster associated with flagella formation and chemotaxis in “*F. myxofaciens*”

**Figure 6:**
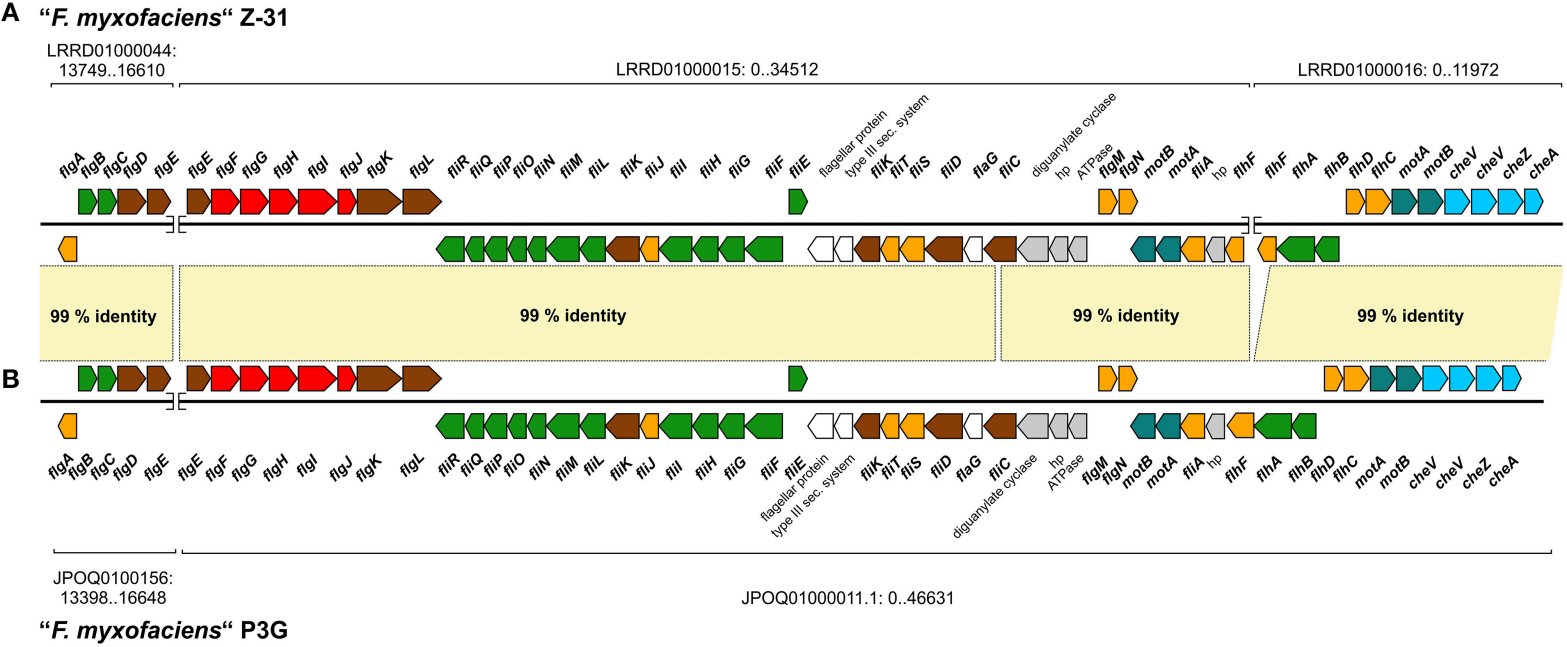
**Homologous gene clusters in both “*F. myxofaciens*” strains predicted to encode proteins involved in the formation of flagella and in chemotaxis**. The gene cluster was found to be distributed over three contigs in in “*F. myxofaciens*” Z-31 **(A)** and over two contigs in “*F. myxofaciens*” P3G **(B)**. Nucleotide accession numbers of the contigs containing the flagella gene cluster and the location of the gene cluster on these contigs are given. Gaps between contigs are indicated by a break of the DNA strand. Genes are colored with respect to the predicted function of their product: flagellum motor, turquoise; flagellum inner membrane structures (basal body and secretion apparatus), green; flagellum outer membrane components, red; flagellum extracellular components (hook and filament), brown; regulation of flagella formation, orange; chemotaxis, light blue; unknown function but associated with flagella, white; not involved in flagella formation, gray. Nucleotide sequence identities between both gene clusters are based on a genome comparison of both “*F. myxofaciens*” strains by blastn and visualization using ACT (light yellow, Carver et al., 2005).

Using the “*F. myxofaciens*” genomes as reference for the blastn-based genome comparison revealed a large genomic region that was absent in the group 2 genomes (Figure 5B, strain Z-31: 470–505 kbp; Figure 5D, strain P3G: 505–550 kbp). This region harbors 35 (Z-31) or 46 (P3G) genes predicted to be involved in flagella formation and chemotaxis while other genes associated with the flagella formation were found to be located in clusters on other contigs. Although the flagella formation and chemotaxis genes are distributed over three contigs in “*F. myxofaciens*” Z-31 and over two contigs in “*F. myxofaciens*” P3G, they appear to be organized in a huge cluster (Figure 6, 51 genes) which was reconstructed based on a comparison with the neutrophilic iron oxidizers *S. lithotrophicus* ES-1 (NC_013959) and *G. capsiferriformans* ES-2 (NC_014394). Pairwise comparison of the reconstructed flagella gene clusters in the “*F. myxofaciens*” strains using ACT revealed the identical order and orientation of the 51 genes as well as an average nucleotide sequence identity of 99% (Figure 6).

The whole genome comparison of all four genomes conducted within Mauve showed that the flanking regions of the flagella gene cluster were syntenic to regions in the group 2 genomes as indicated by the presence of collinear blocks (Supplementary Figure 1). The collinear blocks harbor genes predicted to encode a glutamine-fructose-6-phosphate transaminase in case of the upstream flanking site and an L-threonine-ammonia-ligase in case of the putative downstream flanking site.

The contigs on which the clusters are located were investigated for signatures of HGT in order to infer the putative origin of the flagella gene cluster. However, no transposases, integrases and phage-associated genes were predicted on the corresponding contigs in either of the “*F. myxofaciens*” genomes (Supplementary Table 4) and no variations in G+C content were detected (Figures 5B,D). Therefore, we propose that the absence of the flagella gene cluster in the group 2 strains was more likely the result of gene loss in a common ancestor of both group 2 strains rather than a gene gain by HGT.

##### Gene clusters associated with the utilization of alternative nitrogen sources

**Figure 7:**
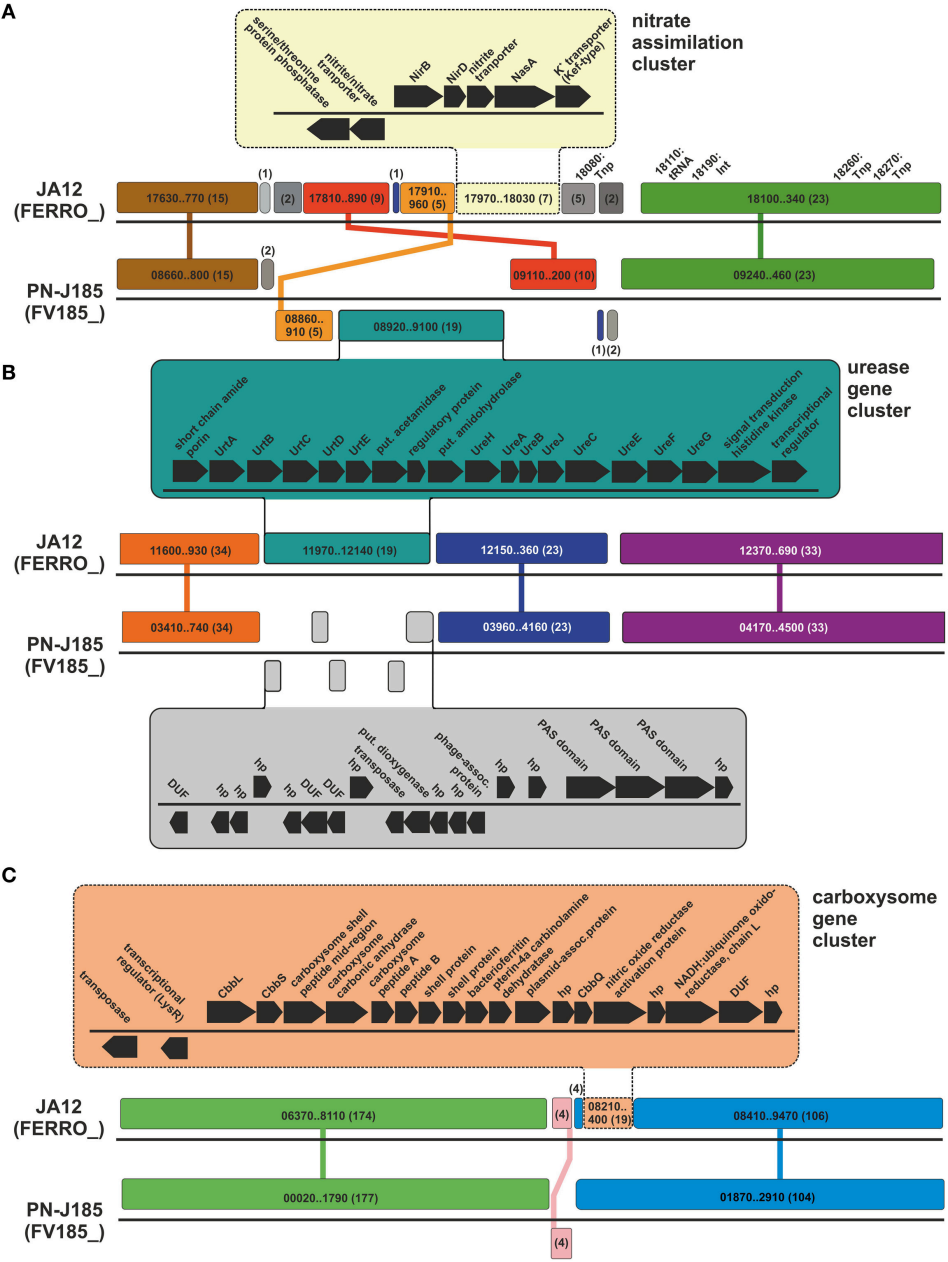
**Homologous genome regions in the group 2 strains JA12 and PN-J185 involved in nitrate assimilation (A), urea utilization (B) and carboxysome formation (C)**. Conserved segments (collinear blocks) were predicted using Mauve (Darling et al., 2010). Collinear blocks between both genomes are shown in the same color and are connected by lines. Locus tags (JA12: FERRO_; PN-J185: FV185_) and number of genes (in parentheses) is given for the collinear blocks. Selected regions containing the nitrate assimilation gene cluster **(A)**, the urea gene cluster (**A**: PN-J185; **B**: JA12) and the carboxysome gene cluster **(C)** are shown in more detail. Nitrite reductase, NirBD; nitrate reductase, NasA; transposase, Tnp; integrase, Int; urea ABC transporter, UrtABCDE; urease, UreABC, urease accessory proteins, UreHJEFG; hypothetical protein, hp; proteins containing a domain of unknown function, DUF; RuBisCO, CbbLS; RuBisCO activating protein, CbbQ.

In the genome of the group 2 strain JA12 a small unique region (Figure 5A: 1760–1780 kbp) was identified that harbors genes predicted to be involved in nitrate assimilation. The genomic neighborhood of this nitrate assimilation gene cluster was compared to the group 2 strain PN-J185 using Mauve (Figure 7A). The nitrate assimilation gene cluster appeared to be integrated in a genomic region that has undergone several events of rearrangements, though this region still consists of the same collinear blocks in both genomes. Three transposases, an integrase and a tRNA gene were predicted downstream of the nitrate assimilation gene cluster suggesting that this cluster was introduced via HGT.

A region unique to the genomes of the group 2 strains was found to harbor the genes associated with the utilization of urea (Figure 5A, JA12: 1140–1150 kbp; Figure 5C, PN-J185: 1550–1560 kbp). Notably, the urease gene cluster is located in different genomic regions in both group 2 genomes which are otherwise characterized by the presence of collinear blocks (Figures 7A,B). However, the urease gene clusters are very similar with regard to gene content, order and orientation. Pairwise comparison of both urease gene clusters visualized using ACT revealed nucleotide sequence identities of up to 81% (Supplementary Figure 2) suggesting that the urease gene clusters in both genomes were derived from a common ancestor. No potential transposases, integrases, or phage-associated genes as signatures of a recent HGT were identified in the genomic neighborhoods. The different location of the homologous gene cluster in both genomes may have been caused by genomic rearrangements in one or even both strains.

A genomic region restricted to the “*F. myxofaciens*” strains contains several of the *nif* -genes predicted to encode the nitrogenase subunits, a molybdenum-iron cofactor biosynthesis protein and the molybdenum uptake ABC transporter (Figure 5D, P3G: 495–505 kbp). Although homologous genes were also identified in “*F. myxofaciens*” Z-31 (Supplementary Table 3B), they were not detectable as discrete genomic regions in the blastn-based whole genome comparison since they were distributed over several small contigs.

##### Gene cluster associated with carboxysome formation in “*F. myxofaciens*” and group 2 strain JA12

The blastn-based whole genome comparison highlighted a region in the genome of the group 2 strain JA12 (Figure 5A: 760–780 kbp) which had matches to both “*F. myxofaciens*” genomes, but not to the genome of group 2 strain PN-J185. This region harbors genes involved in carboxysome formation, a metabolic trait that the group 2 strain JA12 apparently shares only with the “*F. myxofaciens*” strains.

The synteny of the carboxysome gene cluster was compared to the “*F. myxofaciens*” Z-31 using ACT (Supplementary Figure 3A). The gene content and orientation was found to be similar in both genomes, but the level of nucleotide sequence identity varied between the genes encoded in the gene cluster. The genes encoding the large RuBisCO subunit (*cbbL*), the carbonic anhydrase and one of the large carboxysome shell proteins were predicted to share the highest sequence identities of 60–79%. The G+C content of the carboxysome gene clusters in both genomes was found to be slightly higher (JA12: 50%, Z-31: 65%) compared to the average G+C content of the genomes (JA12: 44.5%, Z-31: 54%) (Supplementary Figure 3A). In the genome of group 2 strain JA12 other signatures for HGT were identified including plasmid-associated genes within the gene cluster, two integrase-encoding genes in its downstream region and a tRNA gene and a transposase in its upstream region. However, no potential integration sites were identified in the flanking regions of the carboxysome cluster.

Genome comparison between both group 2 strains using Mauve revealed the location of the carboxysome gene cluster in strain JA12 within a shared collinear block with strain PN-J185 (Figure 7C). Apart from the absence of the carboxysome gene cluster in PN-J185, the adjacent genome regions were found to be syntenic to strain JA12. This impression was furthermore supported by the genome comparison of all four strains using Mauve (Supplementary Figure 3B). While the carboxysome cluster was located on two collinear blocks in the genomes of “*F. myxofaciens*” P3G, “*F. myxofaciens*” Z-31 and group 2 strain JA12, only a small fragment of one these two blocks was present in the genome of PN-J185. This finding together with the slightly higher G+C contents of the carboxysome clusters suggests that the carboxysome cluster may have been acquired by a common ancestor of all “*Ferrovum*” strains, whereas it may have been lost during the genome evolution of group 2 strain PN-J185.

##### Putative genomic islands in the group 2 strain JA12

Blastn-based whole genome comparisons revealed three large DNA segments in the genome of group 2 strain JA12 that had only few matches to the other genomes and that were characterized by abnormally increased G+C contents (Figure 5A: 42–126, 1350–1405, and 1530–1550 kbp). These regions were investigated in some detail for signatures of genomic islands including mobility, conjugation, potential integration sites as well as for their genetic content (Juhas et al., 2009; Bustamante et al., 2012; Acuña et al., 2013).

**Figure 8:**
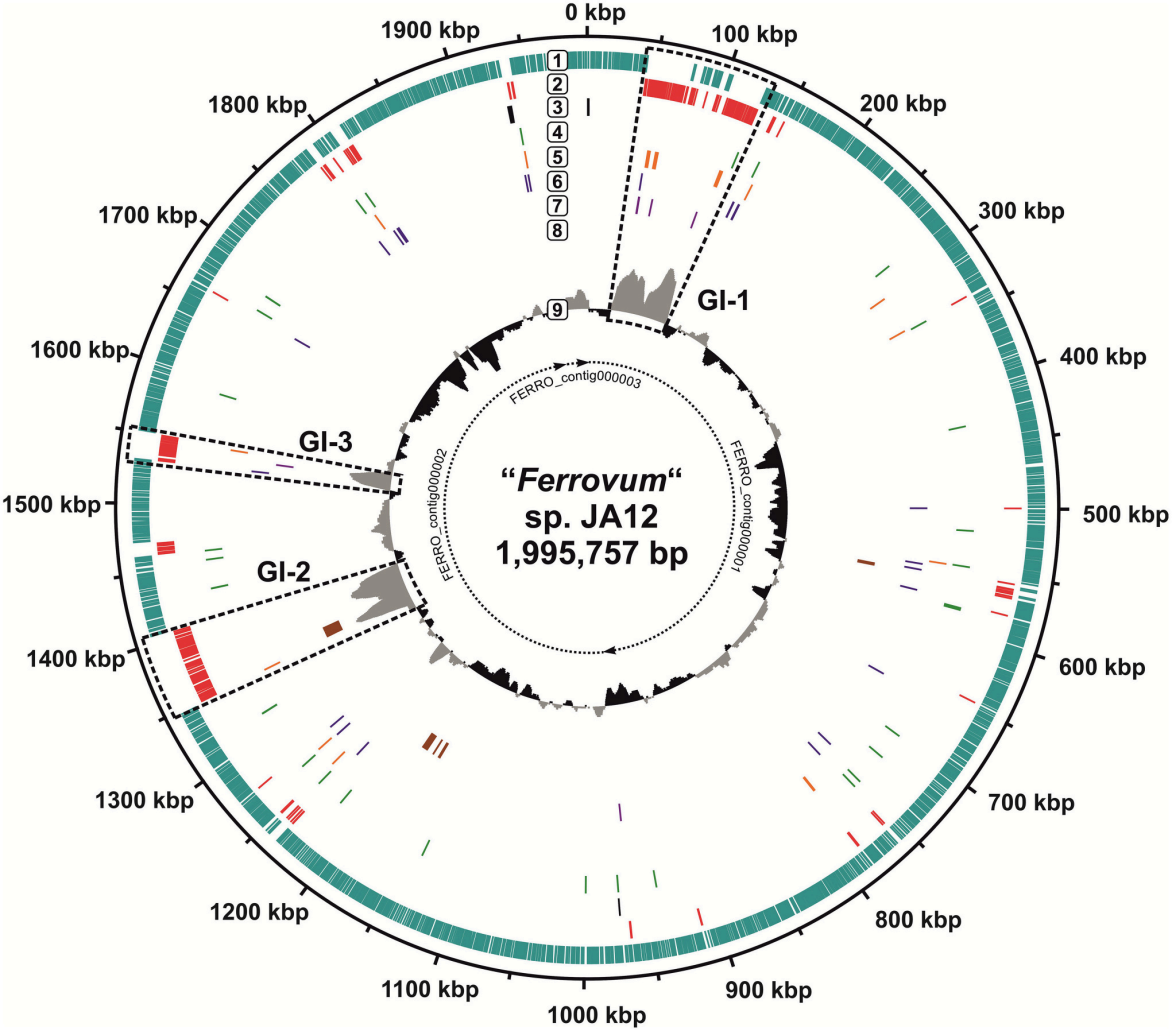
**Potential genomic islands in group 2 strain JA12**. The circular representation of the genome of group 2 strain JA12 was visualized using Artemis (Rutherford et al., 2000) and DNAPlotter (Carver et al., 2009). The borders of the contigs are given in the center (nucleotide accession numbers: FERRO_contig000001, LJWX01000001; FERRO_contig000002, LJWX01000002; FERRO_contig000003, LJWX01000003). The putative genomic islands are named GI-1, GI-2, and GI-3. The rings are numbered from the outside to the inside: 1. coding sequences with normal codon usage (turquoise); 2. putative alien genes (red); 3. rRNA genes (black); 4. tRNA genes (green); 5. predicted integrases (orange); 6. predicted transposases (blue); 7. predicted phage-associated genes (purple); 8. VirB/D4 secretion system (brown); 9. G+C content (gray, above genome average; black, below genome average). The predicted donors and gene functions of the putative alien genes are provided in Supplementary Figure 4 and Supplementary Table 5.

The three regions are enriched in horizontally transferred genes (alien genes) based on the predictions obtained from the application of Sigi-HMM (Figure 8). Putative genomic island 1 (Figure 8: GI-1, 42–126 kbp) is ~85 kbp in size and has an average G+C-content of 56.1%, which is 11% higher than the average G+C-content of the complete genome. The 105 genes located in this genomic region were predicted to encode mobile genetic elements (integrases, phage-associated genes, a transposase, the mobile mystery proteins A and B), tRNAs, hypothetical proteins, glycosyltransferases and enzymes of the nucleotide sugar metabolism, and genes with other predicted functions (Supplementary Table 5A, Supplementary Figure 4). The glycosyltransferases are unique for the genome of group 2 strain JA12 (Supplementary Table 3). The enzymes of the nucleotide sugar metabolism represent duplicate copies of enzymes involved in the formation of the polysaccharide precursors UDP-glucose, TDP-4-dehydro-rhamnose, TDP-rhamnose, TDP-4-dehydro-6-desoxy-glucose, and UDP-N-acetyl-mannosamine (UDP-glucose pyrophosphorylase, dTDP-4-dehydrorhamnose-3,5-epimerase, dTDP-4-dehydrorhamnose reductase, dTDP-glucose 4,6-dehydratase, UDP-N-acetyl-glucosamine 2-epimerase). The majority of genes (91) harbored by GI-1 were predicted to represent alien genes with many of them presumably originating from *Beta*- (31) and *Gammaproteobacteria* (16), and from *Actinobacteria* (9) (Supplementary Table 5A, Supplementary Figure 4). In the right flanking region of GI-1 a tRNA gene marks the transition point from the high G+C content of the island to the approximately average G+C content of the genome (Supplementary Figure 5A: 126 kbp). Upstream to this tRNA gene an integrase-encoding gene is located. Downstream of the tRNA gene a nearly identical direct repeat of a 22 bp-long region of the left flanking region was identified representing potential integration sites of the island.

The second putative genomic island (Figure 8: GI-2, 1350–1405 kbp) is ~56 kbp in size with an average G+C content of 59.2%. This region harbors genes predicted to encode hypothetical proteins, an integrase and a nearly complete set of VirB/D4 proteins of a type IV secretion system (Supplementary Table 5A, Supplementary Figure 4). The *vir*-genes were predicted to be mainly derived from *Betaproteobacteria*. Potential integration sites were identified in the left and right flanking regions of the island (Supplementary Figure 5B). A direct repeat of a 24 bp-long region of the right flanking region was identified in close proximity to an integrase-encoding gene, which is located ~500 bp upstream of the *vir*-genes. Thus, this genomic island shares characteristics with integrative conjugative elements (Wozniak et al., 2009; Bustamante et al., 2012).

The third putative genome island 3 (Figure 8: GI-3, 1530–1550 kbp) has a size of ~20 kbp and exhibits an average G+C content of 55.7, 20% higher than the average for the genome. It is predicted to encode only hypothetical proteins or proteins of unknown function, of which most were predicted to be derived from *Beta*- and *Gammaproteobacteria* (Supplementary Table 5A). Although an integrase was predicted at the right flanking site, no repeat regions were identified at the flanking sites.

The genome of group 2 strain JA12 was found to contain a total of 216 predicted alien genes of which 162 are located on the putative genomic islands. Most of the alien genes were predicted to be derived from *Beta*- and *Gammaproteobacteria* (29 and 18%), but a smaller number of genes was predicted to be derived from other donors including *Actinobacteria* (8%), *Sphingobacteria* (7%), *Bacilli* (6%), and *Bacteroides* (4%) (Supplementary Table 5B). For 46 of the alien genes no donor was predicted using Sigi-HMM.

#### CRISPR/Cas in “*F. myxofaciens*” Z-31: a defense mechanism against foreign DNA

**Figure 9:**
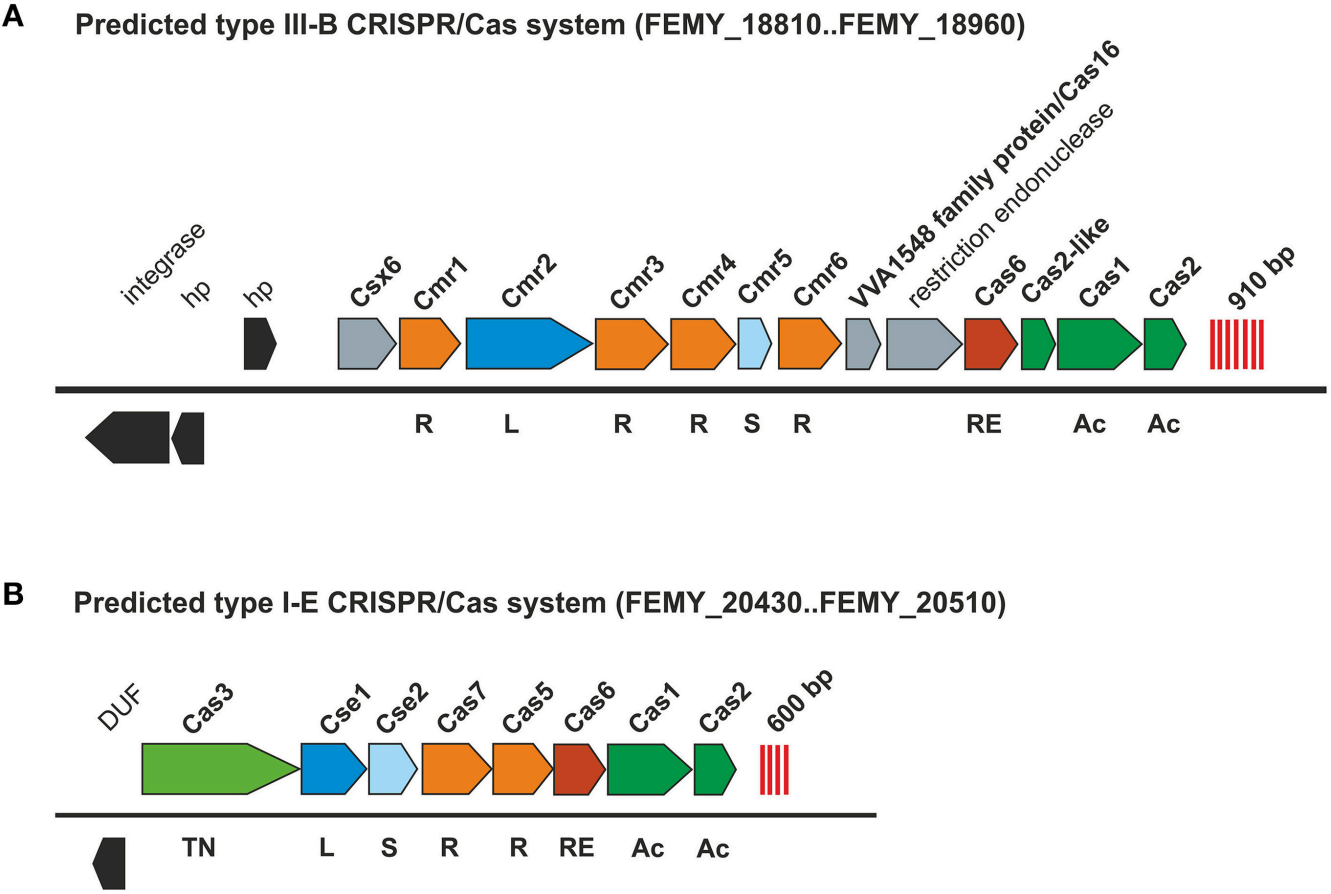
**Predicted defense mechanisms against foreign DNA in the genome of “*F. myxofaciens*” Z-31**. The genome of “*F. myxofaciens*” was predicted to harbor a putative type III-B **(A)** and a presumed type I-E CRISPR/Cas system **(B)**. Predicted CRISPRs are indicated as red lines. Genes encoding homologous proteins have the same color. Cas 1 and 2 (green) are conserved in both systems and are predicted to be involved in the acquisition (Ac) phase recognizing foreign DNA. The Cascade complex is presumed to consist of multiple subunits including a large (L) and small (S) subunit as well as other subunits (R). A specific nuclease (RE) is predicted to be involved in the maturation process of the pre-crRNA (RNA transcript of the CRISPR array) to crRNA of the active Cascade complex. In type I-E Cas3 functions as DNA endonuclease (TN) degrading the foreign DNA bound to the Cascade complex. Genes encoding other Cas-proteins without a specified function are shown in gray while other protein-coding genes are shown in black.

The genome of “*F. myxofaciens*” Z-31 contains two unique regions predicted to encode CRISPR/Cas systems (clustered regularly interspaced palindromic repeats/ CRISPR-associated genes; Figure 5A: 1580–1600 kbp, 1180–1885 kbp; Figure 9). CRISPR/Cas systems have been identified in many bacterial and archaeal genomes and are thought to be part of the host's defense against the invasion of foreign nucleic acids of phages and plasmids (Plagens et al., 2015). The CRISPR/Cas systems in “*F. myxofaciens*” Z-31 were classified as type III-B (Figure 9A) and type I-E (Figure 9B) based on comparison to systems in other bacteria (Makarova et al., 2011). While type I-E systems are described to target DNA, type III-B systems are predicted to target both foreign DNA and RNA. Although only a prophage fragment was identified in “*F. myxofaciens*” Z-31, the presence of the CRISPR/Cas systems strongly suggests that the co-evolution between phage and host may also play an important role in its genome evolution.

## Discussion

### The comparison of their metabolic profiles indicates the existence of group- and strain-specific features

The phylogenetic analysis revealed a diversity among the four “*Ferrovum*” strains that reflects the observed differences of their genome properties and metabolic profiles and, hence, highlights the existence of group- and strain-specific metabolic capacities. The “*F. myxofaciens*” strains (group 1) have larger genomes (>2.5 Mbp) with higher G+C contents (54%) than both group 2 strains (< 2.0 Mbp, < 45%). In this context, the genome of group 2 strain PN-J185 was found to be even smaller (~100 kbp) and to have a lower G+C content (~5%) than that of group 2 strain JA12.

The metabolic profiles of the “*F. myxofaciens*” strains are characterized by the potential to fix molecular nitrogen, to form flagella and to actively move in response to environmental stimuli (chemotaxis). In contrast to this, the group 2 strains appear to be non-motile and they seem to balance their dependence on fixed nitrogen compounds by the potential to use a variety of nitrogen sources such as ammonium, urea and amino acids and, in case of group 2 strain JA12, even nitrate. The ability to use nitrate also indicates a heterogeneity among the group 2 strains, which is further underlined by the absence of the gene repertoire to form carboxysomes in group 2 strain PN-J185. The composition of lipo- and exo-polysaccharides is another distinguishing metabolic feature of the “*Ferrovum*” strains. Although all strains share a common core set of genes encoding enzymes for nucleotide sugar metabolism and glycosyltransferases, each strain also harbors a set of unique additional genes involved in the formation of polysaccharide precursors.

The diversity of their metabolic profiles may explain why “*Ferrovum*” members have been detected in habitats characterized by different geochemical parameters and why they have different macroscopic appearances. For example, the potential to produce biofilms appears to correlate with the presence of the PEP-CTERM exosortase system in the “*F. myxofaciens*” genomes, since this protein-exporting system is known to be associated with biofilm formation (Haft et al., 2006; Craig et al., 2011). In the “*F. myxofaciens*” strains the PEP-CTERM exosortase system may contribute to the massive formation of streamers which presents their most prominent macroscopic feature (Hallberg et al., 2006; Hedrich et al., 2011b; Ziegler et al., 2013; Johnson et al., 2014). This notion is also supported by the high fraction of proteins (17%) within the streamers (Johnson et al., 2014).

Furthermore, the identification of additional Na^+^/H^+^-antiporters and the Kdp-type potassium uptake system in the genomes of the “*F. myxofaciens*” strains suggests a higher pH tolerance since both systems are typically part of the repertoire of extreme acidophiles that cope with acidic environments (Baker-Austin and Dopson, 2007; Slonczewski et al., 2009). At the laboratory scale the type strain “*F. myxofaciens*” P3G grows down to pH 2.2 (Johnson et al., 2014), whereas no cellular activity was observed below pH 2.5 in case of the mixed cultures containing the group 2 strains. Different levels of pH tolerance among the “*Ferrovum*” strains may also explain the reported abundance of “*Ferrovum*” spp. over a pH range of 2.5–3.3 in the Red Eyes mine drainage in the Appalachian Mountains (Pennsylvania, US, Jones et al., 2015).

The *bd*-type quinol oxidase, which is only encoded in the genomes of the group 2 strains, is characterized by a high oxygen affinity (Borisov et al., 2011). It may enable the group 2 strains to thrive under microaerobic conditions like the “*F. myxofaciens*”-related iron oxidizer that was detected in the microaerobic layer of an acidic pit lake in the Iberian Pyrite Belt (Santofimia et al., 2013).

### Genome evolution of the “*Ferrovum*” strains appears to be driven by horizontal gene transfer and genome reduction

A comparison of their genome architectures and the analyses of their genome synteny provide opportunities to infer the potential mechanisms of genome evolution and speciation of the “*Ferrovum*” strains.

#### Horizontal gene transfer

Signatures of horizontal gene transfer (HGT) including transposases, integrases, phage-associated genes, and genes encoding proteins of the type IV secretion system were identified in the genomes of all “*Ferrovum*” strains. The activity of transposases and integrases may have contributed to the observed genomic rearrangements between the genomes of the group 2 strains (i.e., location of the urease gene cluster). A similar observation has been reported for a *Ferroplasma acidarmanus* population in AMD biofilms (Iron Mountain, California, US, Allen et al., 2007). Furthermore, in group 2 strain JA12 transposases and integrases were found to be co-localized with the carboxysome gene cluster and the nitrate assimilation gene cluster suggesting the potential acquisition of genes and metabolic traits via HGT. The extent of HGT events is further suggested by the high number of predicted alien genes within the genomic islands of group 2 strain JA12.

The relevance of genomic islands in mediating HGT in acidophiles has already been described in *A. ferrooxidans* (Cárdenas et al., 2010; Bustamante et al., 2012), *Acidithiobacillus caldus* (Acuña et al., 2013), and *Acidithiobacillus ferrivorans* (González et al., 2014). The genome of the group 2 strain JA12 harbors three potential genomic islands that were identified as large genome regions characterized by their considerably higher G+C contents compared to the genome average, their exclusive presence in the genome of group 2 strain JA12 and their high concentration of putative alien genes. Genomic island 1 harbors numerous accessory genes involved in the formation of precursors for the biosynthesis of cell envelope polysaccharides and an additional set of glycosyltransferases. Since these accessory genes may allow the formation of polysaccharides with altered composition, they may influence the capacity of group 2 strain JA12 to attach to surfaces similar to the assumption made for *A. caldus* (Acuña et al., 2013). Genomic island 2 is presumed to be an integrative conjugative element (ICE) since it encodes a set of genes involved in the type IV secretion system. The putative ICE in group 2 strain JA12 has a size of 56 kbp and contains only hypothetical proteins apart from the type IV secretion system. It is considerably smaller than ICE 1 in *A. ferrooxidans* ATCC 23270 (Bustamante et al., 2012) and in contrast to *A. ferrooxidans* no physiological relevance can be deduced. The identification of signatures of mobility (integrases, putative integration sites, type IV secretion system) on the genomic islands 1 and 2 suggests that they may play a role in promoting genetic exchanges with other community members.

The majority of donors of the predicted alien genes in the group 2 strain JA12 were found to belong to the *Beta*- and *Gammaproteobacteria* which often occur in the microbial communities of AMD habitats (Méndez-García et al., 2015) and which are the closest relatives to “*Ferrovum*” spp. *Beta*- and *Gammaproteobacteria* have also been detected in the original habitats of “*F. myxofaciens*” P3G (Mynydd Parys copper mine effluent, Hallberg et al., 2006) and the other three “*Ferrovum*” strains (lignite mining site in Lusatia, Heinzel et al., 2009a,b). In this context it should be noted that *Acidithiobacillus* spp. were originally assigned to the *Gammaproteobacteria*, whereas they have recently been shown to form the distinct class *Acidithiobacillia* (Williams and Kelly, 2013). Potential betaproteobacterial donors may be other “*Ferrovum*” strains, *Gallionella*-like strains (Bruneel et al., 2006; Heinzel et al., 2009a; Fabisch et al., 2013; Bertin et al., 2011; Liljeqvist et al., 2015), *Sideroxydans*-like strains (Liljeqvist et al., 2015), and strains of the genus *Thiomonas* (Arsène-Ploetze et al., 2010; Slyemi et al., 2011) which have been detected in several AMD habitats. Other predicted donors of alien genes belonging to the *Actinobacteria, Firmicutes* (*Bacilli, Clostridia*), and *Chloroflexi* have also been detected during the original analysis of the diversity of the lignite mining site in Lusatia (Heinzel et al., 2009a). Intriguingly, nearly 12% of the alien genes were predicted to be derived from *Sphingobacteria* or *Bacteroides*, which have not been detected at the lignite mining site (Heinzel et al., 2009a), though this is most likely due to the bias of one of the PCR primers used by Heinzel et al. (2009a) against the 16S rRNA gene of the *Bacteroidetes* (Mühling et al., unpublished results). The more detailed analysis of potential DNA donor-recipient networks may be an interesting prospect for future studies to investigate the evolution of community members in the context of the gene pool of the whole community as has described elsewhere (Dagan, 2011).

Moreover, transduction and phage-host co-evolution evolution appear also to play a role as a means of HGT and genome evolution in “*Ferrovum*.” This is, for example, highlighted by the identification of the prophage fragment and the two CRISPR/Cas systems in “*F. myxofaciens*” Z-31. Evidence for the relevance and rapid course of similar phage-host co-evolution events has already been provided for an AMD biofilm by a metagenomics study which combined the analysis of prophage sequences and spacer sequences of the CRISPR regions in the host genomes (Andersson and Banfield, 2008).

#### Mechanisms of genome reduction

Comparison of the genome architectures of the four strains suggests that the flagella gene cluster may have been lost by both group 2 strains and that the carboxysome gene cluster was lost during the evolution of the group 2 strain PN-J185. Possibly, the *nif* -genes involved in nitrogen fixation and the genes predicted to encode the PEP-CTERM protein export system had a similar fate in both group 2 strains. Apparently, the abandonment of these expensive and dispensable metabolic traits has contributed to the considerable reduction of their genome sizes and to shaping their metabolic profiles. The reduction of genome size in free-living prokaryotes even at the cost of the metabolic versatility has been discussed in the context of increased cellular fitness and efficient use of limited nutrients especially in oligotrophic habitats (Boon et al., 2014; Giovannoni et al., 2014; Martínez-Cano et al., 2015). Several mechanisms of genome reduction have been proposed in other organisms including development of a symbiotic lifestyle, an increased mutation rate (mutator strain hypothesis), streamlining (streamlining hypothesis) or as result of community-dependent adaptations (Black Queen Hypothesis) (Morris et al., 2012; Martínez-Cano et al., 2015).

Genome reduction during host-adaption of obligate symbionts has often been reported to be accompanied by the loss of biosynthetic genes involved in amino acid, fatty acid, cofactor and vitamin biosynthesis or of genes involved in DNA repair (D'souza et al., 2014; Martínez-Cano et al., 2015; Nelson and Stegen, 2015). Neither of the group 2 strains harbors these signatures. Instead they exhibit the potential to synthesize all amino acids, fatty acids and cofactors, and harbor a large repertoire of genes predicted to be involved in DNA repair.

Another potential driving force of genome reduction is the abandonment of genes with low contribution to cellular fitness (Marais et al., 2008). Strains that have higher mutation rates due to prior loss of DNA repair systems, are then favored during the colonization of novel ecological niches (mutator strain hypothesis). Since all “*Ferrovum*” strains harbor similar gene repertoires associated with DNA repair, the mutator strain hypothesis does also not provide an explanation for the genome reduction observed in the group 2 strains.

The streamlining hypothesis asserts genome reduction as an effect of natural selection brought about by increased cellular economization (Mira et al., 2001; Dufresne et al., 2005; D'souza et al., 2014). Examples of genome streamlining are found among bacteria in oceanic habitats (*Prochlorococcus*, Dufresne et al., 2003; SAR11 group, Grote et al., 2012) where genome reduction is presumed to be an adaptation to limited nutrient availability, in particular to inorganic phosphate (Paytan and McLaughlin, 2007). The inorganic phosphate concentration is also extremely low in AMD habitats (Walton and Johnson, 1992). Thus, the genome reduction of the group 2 strains appears to be advantageous since it reduces their inorganic phosphate requirement. However, the streamlining hypothesis also states that the maintenance of a functional diversity represents another concept to successfully thrive in an ecological niche, and thereby addresses the observation that often not all microorganisms in a habitat reduce their genome size (Giovannoni et al., 2014). This may explain, why the “*F. myxofaciens*” strains have larger genomes despite being confronted with similar nutrient limitations.

The Black Queen Hypothesis describes genome reduction as a means of adaptation in dependence of the metabolic functions that are harbored by the microbial community (Morris et al., 2012). Thus, some community members lose metabolic functions that are dispensable for individual members as long as the function remains active in the community and the product of the metabolic function is leaked into the community in sufficient amounts. This concept provides an explanation as to why some essential functions (i.e., nitrogen fixation, detoxification) are partitioned in microbial communities and why often only a small fraction of community members harbors these functions (Morris et al., 2012, 2014; Martínez-Cano et al., 2015). According to this concept genome evolution by genome reduction in the group 2 strains can be placed into the context of the potential functional network of the AMD community as illustrated by the following examples.

In AMD communities nitrogen fixation appears to be restricted to a few community members (Méndez-García et al., 2015) including diazotrophic bacteria such as “*F. myxofaciens*” (Johnson et al., 2014), *Leptospirillum ferrodiazotrophum* (Tyson et al., 2004), or *A. ferrooxidans* (Valdés et al., 2008). Given that fixed nitrogen compounds for the growth of the group 2 strains were produced in sufficient amounts by diazotrophic community members, losing the ability to fix molecular nitrogen became beneficial for two reasons. First, the group 2 strains save energy required to maintain the nitrogen fixation machinery and to fix nitrogen. Second, the community benefits from the genome reduction of some community members due to their reduced nutrient requirements and, thus, the economization of the whole community nutrient resources (Morris et al., 2012, 2014). Based on their metabolic potential the “*F. myxofaciens*” strains may provide both ammonium via nitrogen fixation and amino acids via the PEP-CTERM protein export system for the group 2 strains to thrive on. A similar role allocation has been discussed for *Ferroplasma* strains utilizing amino acids and ammonium taken up from the environment, and *L. ferrodiazotrophum* fixing molecular nitrogen in an AMD biofilm (Tyson et al., 2004, 2005).

The possible abandonment of motility, chemotaxis and the PEP-CTERM protein export system (biofilm formation) in both group 2 strains may have followed a similar course. These metabolic traits are advantageous during the early stages of the colonization of new habitats, which may be accomplished by “*F. myxofaciens*” strains as has been reported for an acid mine stream in Wales (Mynydd Parys, Kay et al., 2013). Thus, while the “*F. myxofaciens*” strains appear to maintain a gene repertoire enabling them to colonize new habitats as pioneers, the group 2 strains may belong to later colonizers, but then benefit from their smaller genome size.

## Concluding remarks

This comparative genome study advances our current knowledge of the taxonomic and metabolic diversity of “*Ferrovum*” spp. It also provides an extended framework for assigning sequence reads derived from (environmental) metagenomics studies to these strains and close relatives, forming a basis to investigate the ecological functions of “*Ferrovum*” members in the microbial networks of AMD habitats.

New insights were also gained into the mechanisms of genome evolution and speciation. Apparently, horizontal gene transfer mediated by conjugation, transduction and transfer of genomic islands together with genome reduction either as potential community-adaptive event or as the consequence of genome streamlining have driven diversification of the “*Ferrovum*” strains. This evolutionary episode finally resulted in the speciation of the motile diazotrophic “*F. myxofaciens*” strains and the non-motile group 2 strains with smaller genomes. Habitat-driven genome evolution will be addressed in future studies using the genomes of the “*Ferrovum*” strains derived from the lignite mining site as reference for comparison to metagenomic reads of the same habitat, an approach that has already proved suitable for other AMD microorganisms including *F. acidarmanus* (Allen et al., 2007), *Sulfolobus* spp. (Justice et al., 2014) and *A. ferrivorans* (González et al., 2014).

## Author contributions

The study was proposed by SU, DH, MM, and MS. JT enriched strain PN-J185 from AMD water samples. SU cultivated strain PN-J185 and extracted genomic DNA for sequencing. AP and RD planned genome sequencing and AP conducted the genome sequencing, assembly and automated annotation of the genomes of strains Z-31 and PN-J185. The comparative study was conducted by SU under supervision of DH. CG supported SU during application of the bioinformatics tools. SU wrote the manuscript. DH, MM, and MS contributed to the final manuscript by critical revision. All authors read and approved the manuscript.

### Conflict of interest statement

The authors declare that the research was conducted in the absence of any commercial or financial relationships that could be construed as a potential conflict of interest.
