# Supplementary material for: Gene Loss and Horizontal Gene Transfer Contributed to the Genome Evolution of the Extreme Acidophile “Ferrovum”
Source: Front Microbiol. 2016 May 31;7:797. doi: 10.3389/fmicb.2016.00797 (PMC4886054; doi:10.3389/fmicb.2016.00797)
Supplement: Supplementary file 1 [file Table1.pdf]

Supplementary Material

Gene Loss and Horizontal Gene Transfer Contributed to the Genome Evolution of the Extreme Acidophile "*Ferrovum*"

Sophie R. Ullrich*, Carolina González, Anja Poehlein, Judith S. Tischler, Rolf Daniel, Michael Schlömann, David S. Holmes, Martin Mühling*

*** Correspondence:** martin.muehling@ioez.tu-freiberg.de, sophie.ullrich@ioez.tu-freiberg.de

Supplementary Table 1. Nucleotide accession numbers of genomes used in the comparative study.

Name of the microorganism	Nucleotide accession number
"*Ferrovum myxofaciens*" P3G	NZ_JPOQ01000000
"*Ferrovum myxofaciens*" Z-31	NZ_LRRD00000000
"*Ferrovum*" sp. JA12	NZ_LJWX00000000
"*Ferrovum*" sp. PN-J185	NZ_LQZA00000000
Mariprofundus ferrooxydans PV-1	NZ_AATS00000000
Mariprofundus ferrooxydans M34	NZ_ARAU00000000
Rhodopseudomonas palustris TIE-1	NC_011004
Rhodomicrobium vannielii ATCC 17100	NC_014664
Paracoccus denitrificans PD1222	NC_008686-8
Acidiphilium cryptum JF-5	NC_009484
Geobacter metallireducens GS-15	NC_007517
Geobacter uraniireducens Rf4	NC_009483
Alcanivorax borkumensis SK2	NC_008260
Escherichia coli K12 substr. MG1655	NC_000913
Nitrosococcus oceani ATCC 19707	NC_007484
Acidithiobacillus caldus SM-1	NC_015850
Acidithiobacillus ferrivorans SS3	NC_015942
Acidithiobacillus ferrooxidans ATCC 23270	NC_011761
Nitrosospira multiformis ATCC 25196	NC_007614
Nitrosomonas europaea ATCC 19718	NC_004757
Nitrosomonas eutropha C71	NC_008344
Thiobacillus denitrificans ATCC 25259	NC_007404
Pseudogulbenkinia strain NH8B	NC_016002
Chromobacterium violaceum ATCC 12472	NC_005085
Burkholderia multivorans ATCC 17616	NC_010805
Acidovorax ebreus TPSY	NC_011992
Gallionella capsiferriformans ES-2	NC_014394
Sideroxydans lithotrophicus ES-1	NC_013959
Azospira suillum PS	NC_016616
