# Supplementary material for: Gene Loss and Horizontal Gene Transfer Contributed to the Genome Evolution of the Extreme Acidophile “Ferrovum”
Source: Front Microbiol. 2016 May 31;7:797. doi: 10.3389/fmicb.2016.00797 (PMC4886054; doi:10.3389/fmicb.2016.00797)
Supplement: Supplementary file 6 [file DataSheet1.pdf]

Supplementary Material

Gene Loss and Horizontal Gene Transfer Contributed to the Genome Evolution of the Extreme Acidophile "*Ferrovum*"

Sophie R. Ullrich*, Carolina González, Anja Poehlein, Judith S. Tischler, Rolf Daniel, Michael Schlömann, David S. Holmes, Martin Mühling*

*** Correspondence:** martin.muehling@ioez.tu-freiberg.de, sophie.ullrich@ioez.tu-freiberg.de

Supplementary Data 1: Predicted metabolic potential of the four "*Ferrovum*" strains (detailed description of Figure 3 in the main text).

Carbon fixation and central carbon metabolism. All "*Ferrovum*" strains were predicted to fix carbon dioxide *via* the Calvin-Benson-Bassham cycle (CBB) using the energy derived from aerobic ferrous iron oxidation. With exception of the group 2 strain PN-J185, the other "*Ferrovum*" strains were predicted also to utilize bicarbonate as carbon source in the carboxysome (CBS). The carbon fixation product 3-phosphoglycerate (G3P) was predicted to be further metabolized either through the pentose phosphate pathway to generate phosphoribosyl pyrophosphate (PRPP), which is a precursor for the biosynthesis of purines and pyrimidines, or by the glycolysis and gluconeogenesis. The conversion of G3P by the glycolysis was predicted to lead to the formation of acetyl-CoA which then either serves as precursor for the biosynthesis of fatty acids, or is further metabolized by the tricarboxylic acid (TCA) cycle to precursors of the amino acid biosynthesis. All strains were predicted to harbor the complete enzyme repertoire of the TCA cycle. Furthermore, G3P was predicted to be metabolized *via* gluconeogenesis to glucose-6-phosphate (Glc) that may further be converted to nucleotide-activated monosaccharides (NDP-monosaccharides) by the amino sugar and nucleotide sugar metabolism. NDP-monosaccharides were predicted to serve as precursors for the biosynthesis of the cell envelope (inner membrane, IM; periplasm, P; outer membrane, OM) polysaccharides peptidoglycan, lipopolysaccharides and exopolysaccharides. Alternatively to the formation of biomass, organic carbon compounds may be stored either as glycogen in case of the "*F. myxofaciens*" strains or, as polyhydroxybutyrate (PHB) in case of the OUT-2 strains. (See also Supplementary Table 3A)

Nitrogen assimilation. All four strains were predicted to take up ammonium *via* an Amt family transporter and to transfer it to glutamate *via* a glutamine synthetase (GlnA). The "*F. myxofaciens*" strains were predicted to also fix molecular nitrogen *via* the nitrogenase using ferredoxin (fd) as electron donor for the reduction of nitrogen to ammonium. The group 2 strains appear to utilize urea, which is predicted to taken up *via* the urea ABC transporter (Urt) and hydrolyzed in the cytoplasm *via* the enzyme urease (Ure). The generated ammonium may be transferred to glutamate while group

2 strain JA12 potentially fixes the bicarbonate that is also released during urea hydrolysis, through the activity of the carbonic anhydrase and the RuBisCO within the carboxysome. Furthermore, the group 2 strain JA12 appears to utilize nitrate as potential nitrogen source using predicted assimilatory nitrate (NasA) and nitrite (NirBD) reductases. (See also Supplementary Table 3B).

Energy metabolism. The "*Ferrovum*" strains appear to oxidize ferrous in the outer membrane *via* a Cyc2-like high molecular mass cytochrome *c* and to transfer electrons downhill to the cbb_3-type cytochrome *c* oxidase *via* soluble *c*-type cytochromes (Cyt*C*) in the periplasm. The cbb_3-type cytochrome *c* oxidase and the bo_3-type quinol oxidase are furthermore predicted to pump protons outside the cell driven by the downhill electron transfer to oxygen. The natural proton gradient across the membranes is predicted to drive the ATP synthesis at ATP synthase (ATP synth.) and the uphill electron transfer from ferrous iron to NAD^+ *via* the bc_1 complex and the NADH dehydrogenase (NADH dh) in order to generate reduction equivalents for biosyntheses. The NADH dehydrogenase uses quinol (QH_2) as electron donor. Apart from the bc_1 complex the succinate dehydrogenase (succ. dh) may also channel electrons into the quinol pool. The group 2 strains were predicted to harbor a second quinol oxidoreductase of the *bd*-type apart from the bo_3-type quinol oxidase. Furthermore, the group 2 strains were predicted to encode a formate dehydrogenase (formate dh) using an unknown electron acceptor/donor couple ($donor_{red}$/$donor_{ox}$). The genomes of the "*F. myxofaciens*" strains were predicted to encode a hydrogen-evolving hydrogenase. (See also Supplementary Table 3C)

Cell mobility and chemotaxis. Apparently, the "*F. myxofaciens*" strains harbor the genes required for the formation of flagella and chemotaxis presumably enabling these strains to move in response to certain stimuli. These may be sensed by one of the predicted chemotaxis sensors (Mcp, methyl-accepting chemotaxis sensory transducer; Aer, aerotaxis receptor). The signal is further transduced *via* the predicted two-component system sensor histidine kinases (CheW, CheA) to the two-component system response regulator CheY. FliGMN presumably mediates the signal from CheY to the flagella motor (MotAB), thereby inducing flagella rotation. The chemotaxis proteins CheVBDR were predicted to be involved in the regulation feedback loop. (See also Supplementary Table 3D).

Acid stress management. "*Ferrovum*" strains have to cope with the natural proton gradient between the acidic environment and the assumed circum neutral pH in the cytoplasm. In order to prevent the uncontrolled influx of protons the group 2 strains were predicted to increase the hydrophobicity of their cellular membranes by incorporation of cyclopropane fatty acyl phospholipids using the cyclopropane fatty acyl phospholipid synthase (Cfa). All "*Ferrovum*" strains were furthermore predicted to inhibit the uncontrolled proton influx by maintaining a reversed (inside positive) membrane potential presumably accomplished by the increased uptake of potassium ions *via* Kef-type potassium transporter (Kef). The "*F. myxofaciens*" were predicted to additionally harbor genes encoding a predicted potassium import ATPase (Kdp). Proton pumping activity of respiratory complexes driven by the downhill electron transfer may be involved in coping with an increase of the intracellular proton concentration. Apparently, the "*F. myxofaciens*" additionally use sodium/proton antiporters (NhaD) to extrude excess protons. An alternative strategy could be the buffering of the

intracellular pH by the decarboxylation of arginine (Adi), aspartate (PanD) and phosphatidyl serine (Psd), and the synthesis of polyamines which is presumed to be employed by all "*Ferrovum*" strains. The group 2 strains may additionally benefit from the buffering capacity of ammonia derived from the urea hydrolysis. (See also Supplementary Table 3E)

Coping with high metal concentrations. All "*Ferrovum*" strains were predicted to use cation/multidrug transporters of the RND family to cope with the high metal ion concentrations consisting of a predicted RND pump (RND), two membrane fusion proteins (MFP) and an outer membrane protein (Omp). However, they appear to use specific copper efflux systems. While "*F. myxofaciens*" strains were predicted to export copper ions *via* the Cus system consisting of a pump (CusA), the channel-forming membrane fusion proteins (CusB), the outer membrane protein (CusC) and the small periplasmic protein CusF, the group 2 strains may instead use the predicted copper-exporting ATPase CopA. (See also Supplementary Table 3E)

Oxidative stress management. All "*Ferrovum*" strains seem to use a superoxide dismutase (Sod) to convert superoxide radicals to hydrogen peroxides. The "*F. myxofaciens*" strains were predicted to use rubrerythrin to detoxify the generated hydrogen peroxide while the group 2 strains were predicted to use thiol peroxidases (thiol perox.) or ferritins. All "*Ferrovum*" strains may use the thioredoxin (TrxA)/ thioredoxin reductase (TrxB)-dependent system to repair oxidative damaged proteins. TrxB was thereby predicted to restore the original redox state to the oxidized thioredoxin (TrxA'). Additionally, the predicted thiol:disulfide interchange proteins DsbA and DsbD could serve a similar purpose for proteins in the periplasm. A methionine sulfoxide reductase (MrsAB) for the repair of oxidatively damaged methionine residues was only predicted in the "*F. myxofaciens*" strains. The redox state of oxidatively damaged lipids may be restored in all strains by the peroxiredoxin (AhpC)/alkyl hydroxide peroxidase (AhpF)-dependent system. All four strains were predicted to harbor a similar repertoire of genes involved in DNA repair including base and nucleotide excision repair and the mismatch repair. (See also Supplementary Table 3E)
