# Supplementary material for: Gene Loss and Horizontal Gene Transfer Contributed to the Genome Evolution of the Extreme Acidophile “Ferrovum”
Source: Front Microbiol. 2016 May 31;7:797. doi: 10.3389/fmicb.2016.00797 (PMC4886054; doi:10.3389/fmicb.2016.00797)
Supplement: Supplementary file 7 [file Image1.pdf]

Supplementary Material

Gene Loss and Horizontal Gene Transfer Contributed to the Genome Evolution of the Extreme Acidophile "*Ferrovum*"

S.R. Ullrich, C. González, A. Poehlein, J.S. Tischler, R. Daniel, *et al.*

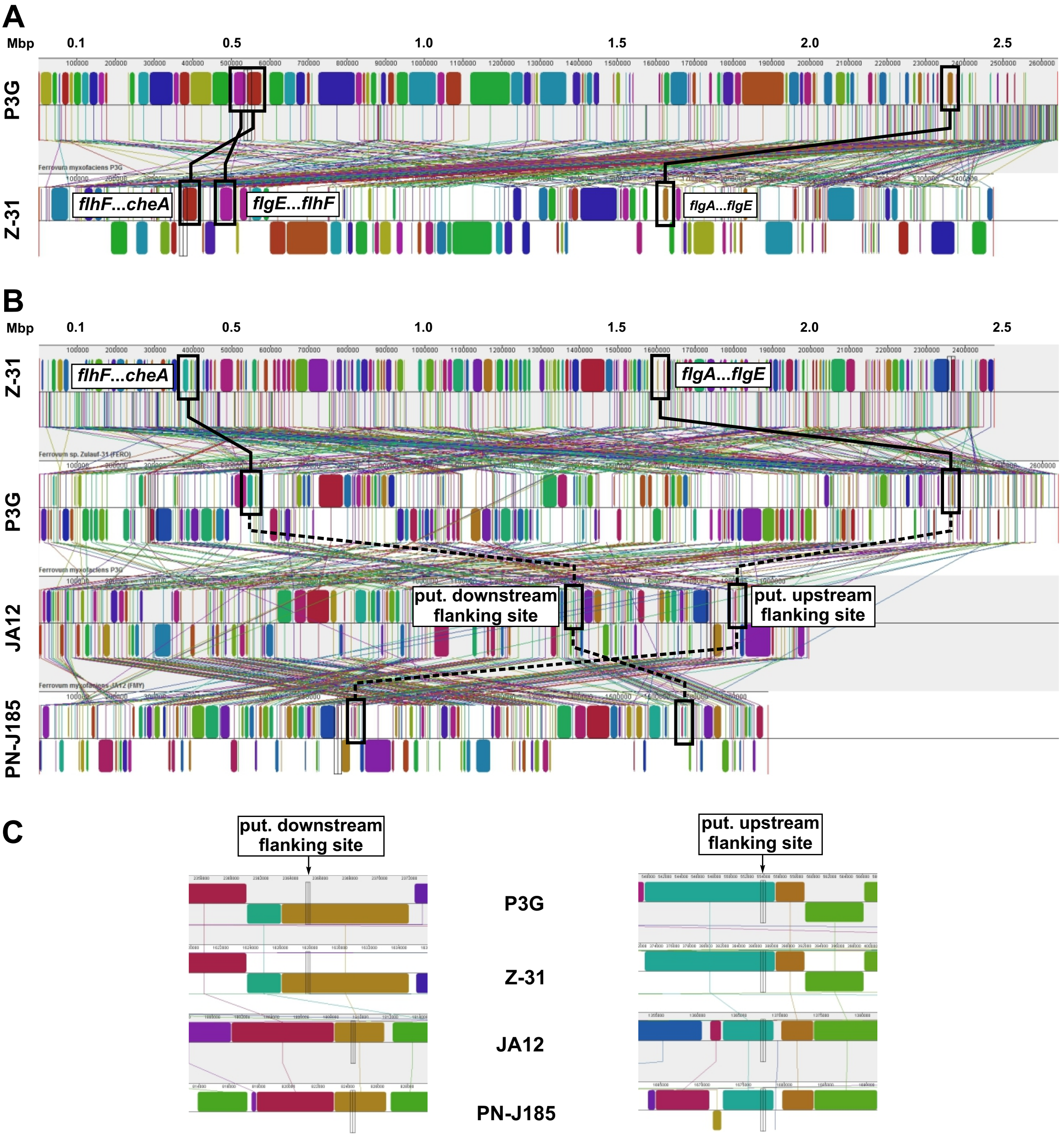

Supplementary Figure 1. Whole genome comparison of the four "*Ferrovum*" genomes conducted and visualized using Mauve (Darling *et al.*, 2010). (A) The collinear blocks corresponding with the flagella gene cluster are highlighted in the whole genome comparison of the "*F. myxofaciens*" strains P3G and Z-31. (B) The collinear blocks corresponding to the upstream and downstream region of the flagella gene cluster are indicated in the whole genome comparison of all four "*Ferrovum*" strains. In the group 2 genomes (JA12, PN-J185) these blocks are smaller due to the absence of the flagella genes. (C) The putative up- and downstream regions of the flagella gene cluster are shown enlarged. The location of genes predicted to encode the glutamine-fructose-6-phosphate transaminase (putative upstream flanking site) and the L-threonine-ammonia-ligase (putative downstream flanking site) is indicated by black boxes in the collinear blocks in the four genomes.
