# Supplementary material for: Gene Loss and Horizontal Gene Transfer Contributed to the Genome Evolution of the Extreme Acidophile “Ferrovum”
Source: Front Microbiol. 2016 May 31;7:797. doi: 10.3389/fmicb.2016.00797 (PMC4886054; doi:10.3389/fmicb.2016.00797)
Supplement: Supplementary file 8 [file Image2.pdf]

Gene Loss and Horizontal Gene Transfer Contributed to the Genome Evolution of the Extreme Acidophile "*Ferrovum*"

S.R. Ullrich, C. González, A. Poehlein, J.S. Tischler, R. Daniel, *et al.*

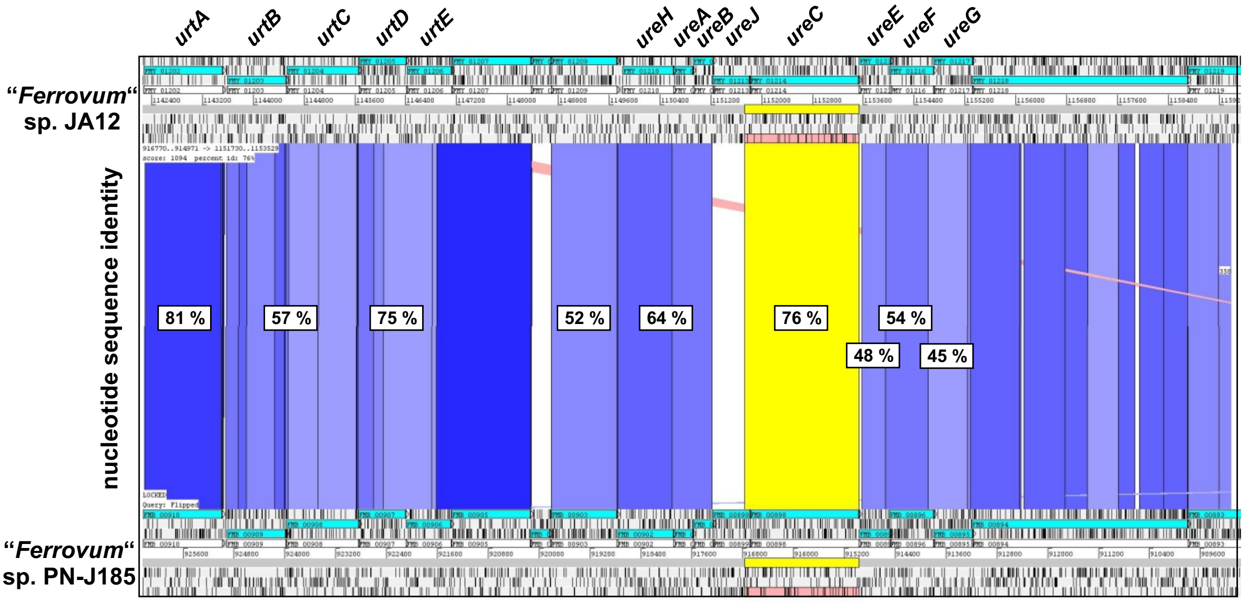

Supplementary Figure 2. Synteny of the urease gene cluster in the group 2 strains JA12 and PN-J185. The genome comparison by tblastx (DoubleACT) was visualized using ACT (Carver *et al.*, 2005) with the sequence of group 2 strain PN-J185 being flipped to facilitate analysis. The predicted gene function and the nucleotide sequence identities of matches are indicated by the intensity of the blue color (the higher the color intensity the higher the sequence intentity). Blue color represents reverse matches which are matches to the opposite strand in the other genome. The match of the *ureC*-gene encoding the largest subunit of the urease is highlighted in yellow.

DoubleACT, http://www.hpa-bioinfotools.org.uk/pise/double_act.html
