# Supplementary material for: Gene Loss and Horizontal Gene Transfer Contributed to the Genome Evolution of the Extreme Acidophile “Ferrovum”
Source: Front Microbiol. 2016 May 31;7:797. doi: 10.3389/fmicb.2016.00797 (PMC4886054; doi:10.3389/fmicb.2016.00797)
Supplement: Supplementary file 9 [file Image3.pdf]

Supplementary Material

Gene Loss and Horizontal Gene Transfer Contributed to the Genome Evolution of the Extreme Acidophile "*Ferrovum*"

S.R. Ullrich, C. González, A. Poehlein, J.S. Tischler, R. Daniel, *et al.*

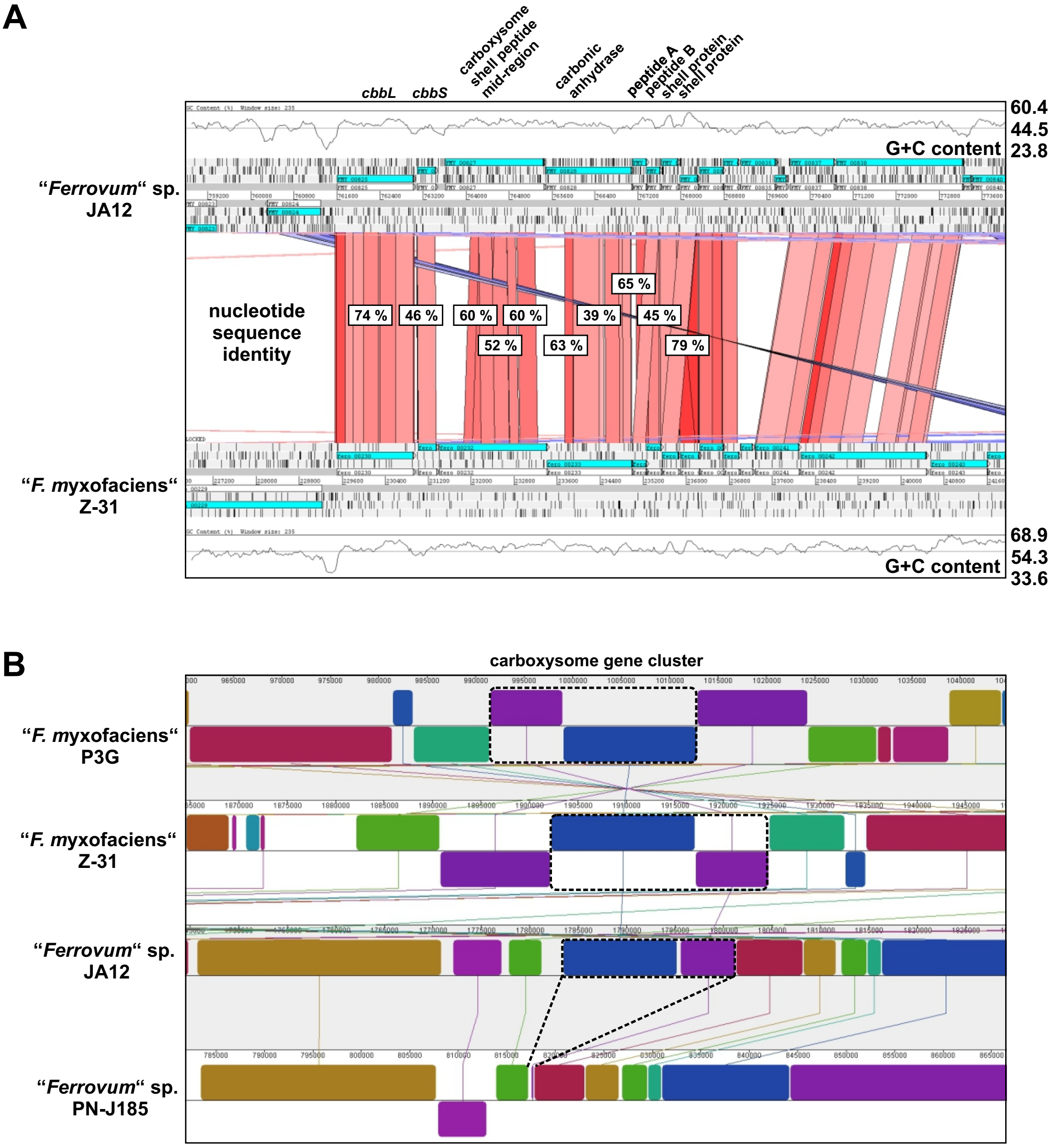

Supplementary Figure 3. Carboxysome gene cluster in the "*F. myxofaciens*" strains and group 2 strain JA12. (A) The synteny of the gene cluster in group 2 strain JA12 and in "*F. myxofaciens*" Z-31 was determined by whole genome comparison using tblastx (DoubleACT) and visualized using ACT (Carver *et al.,* 2005). Predicted gene functions, nucleotide sequence identity of these genes and G+C content of the genome region are shown. (B) The whole genome comparison of all genomes using Mauve indicated the location of the carboxysome gene cluster on two collinear blocks (blue and purple). In the genome of group 2 strain PN-J185 only a little fragment of the purple collinear block is left while the major part of this block and the complete blue block are absent suggesting that the gene cluster was lost during evolution.

DoubeACT, http://www.hpa-bioinfotools.org.uk/pise/double_act.html
