# Supplementary material for: Gene Loss and Horizontal Gene Transfer Contributed to the Genome Evolution of the Extreme Acidophile “Ferrovum”
Source: Front Microbiol. 2016 May 31;7:797. doi: 10.3389/fmicb.2016.00797 (PMC4886054; doi:10.3389/fmicb.2016.00797)
Supplement: Supplementary file 10 [file Image4.pdf]

Gene Loss and Horizontal Gene Transfer Contributed to the Genome Evolution of the Extreme Acidophile "*Ferrovum*"

S.R. Ullrich, C. González, A. Poehlein, J.S. Tischler, R. Daniel, *et al.*

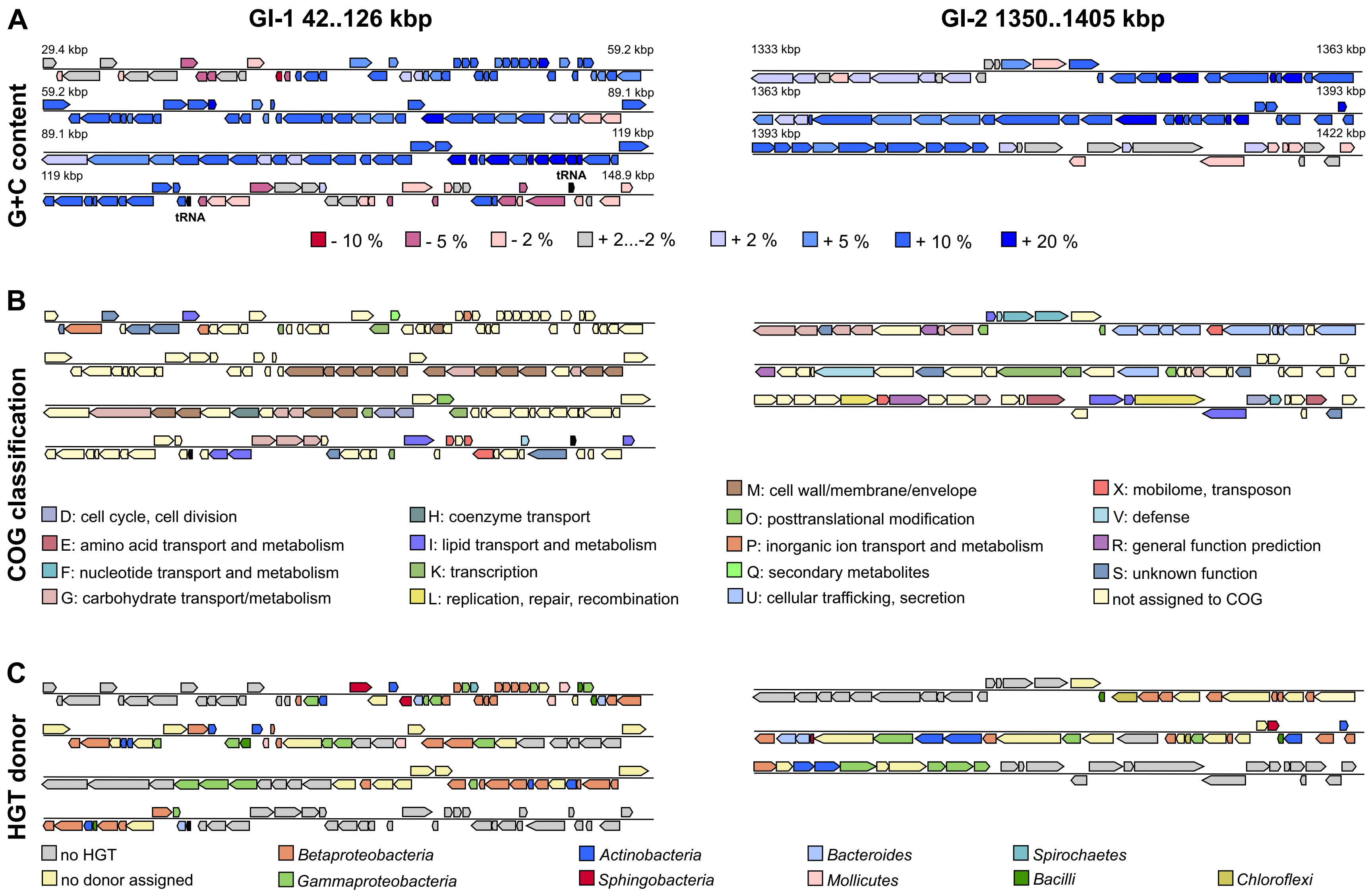

Supplementary Figure 4. Predicted genomic islands 1 and 2 in group 2 strain JA12. The genome regions harboring the predicted genomic islands 1 (GI-1) and 2 (GI-2) are colored according to their G+C content (A), COG classification (B) and the predicted donor of putative horizontally transferred genes (C). G+C content and COG coloring are based on predictions of the IMG/ER pipeline (9 September 2015) (Markowitz *et al.,* 2009, Markowitz *et al.,* 2014). The donors of predicted alien genes putatively acquired *via* horizontal gene transfer (HGT) were predicted using Sigi-HMM (Waack *et al.,* 2006). A detailed summary of all predicted alien genes within genomic islands and other regions (not shown here) is given in Supplementary Table 5.
