# Supplementary material for: Gene Loss and Horizontal Gene Transfer Contributed to the Genome Evolution of the Extreme Acidophile “Ferrovum”
Source: Front Microbiol. 2016 May 31;7:797. doi: 10.3389/fmicb.2016.00797 (PMC4886054; doi:10.3389/fmicb.2016.00797)
Supplement: Supplementary file 11 [file Image5.pdf]

Supplementary Material

Gene Loss and Horizontal Gene Transfer Contributed to the Genome Evolution of the Extreme Acidophile "*Ferrovum*"

S.R. Ullrich, C. González, A. Poehlein, J.S. Tischler, R. Daniel, *et al.*

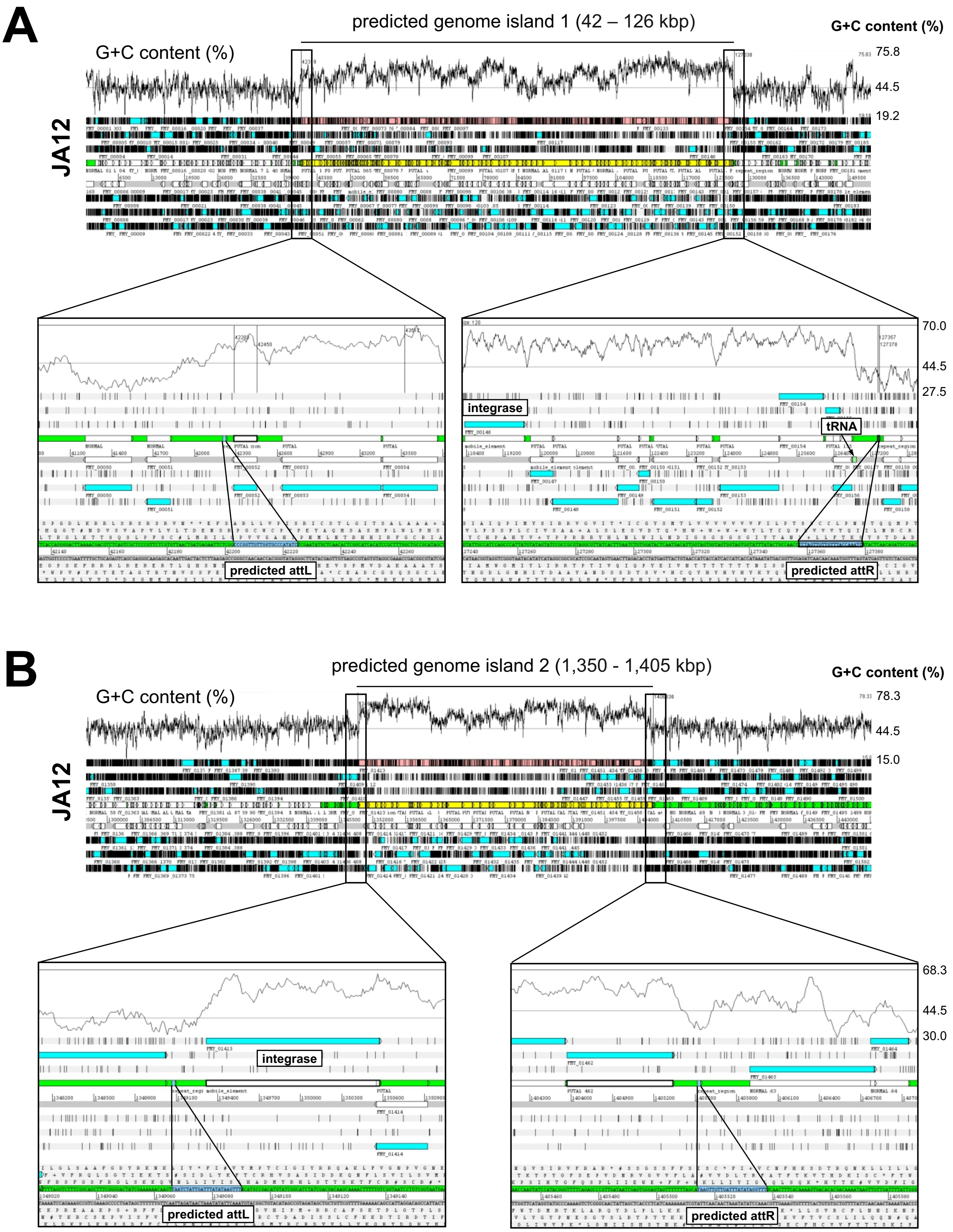

Supplementary Figure 5. Flanking sites of the predicted genomic islands 1 and 2 in group 2 strain JA12. The flanking sites in genomic island 1 (A) and 2 (B) were assigned to regions characterized by abrupt changes of G+C content. The visualization and manual inspection for the presence of direct repeats (left flanking site, *attL*; right flanking site, *attR*; blue) were conducted using Artemis (Rutherford *et al.,* 2000).
